# Selection for stress tolerance and longevity in *Drosophila melanogaster* have strong impacts on microbiome profiles

**DOI:** 10.1038/s41598-024-68753-5

**Published:** 2024-08-01

**Authors:** Torsten Nygaard Kristensen, Anna A. Schönherz, Palle Duun Rohde, Jesper Givskov Sørensen, Volker Loeschcke

**Affiliations:** 1 Department of Chemistry and Bioscience, Aalborg University, Fredrik Bajers Vej 7H, 9220 Aalborg, Denmark; 2 Department of Animal and Veterinary Sciences, Aarhus University, Tjele, Denmark; 3 Department of Health Science and Technology, Aalborg University, Aalborg, Denmark; 4 Department of Biology, Aarhus University, Aarhus, Denmark

**Keywords:** Ecology, Evolution, Genetics, Microbiology

## Abstract

There is experimental evidence that microbiomes have a strong influence on a range of host traits. Understanding the basis and importance of symbiosis between host and associated microorganisms is a rapidly developing research field, and we still lack a mechanistic understanding of ecological and genetic pressures affecting host-microbiome associations. Here *Drosophila melanogaster* lines from a large-scale artificial selection experiment were used to investigate whether the microbiota differ in lines selected for different stress resistance traits and longevity. Following multiple generations of artificial selection all selection regimes and corresponding controls had their microbiomes assessed. The microbiome was interrogated based on 16S rRNA sequencing. We found that the microbiome of flies from the different selection regimes differed markedly from that of the unselected control regime, and microbial diversity was consistently higher in selected relative to control regimes. Several common *Drosophila* bacterial species showed differentially abundance in the different selection regimes despite flies being exposed to similar environmental conditions for two generations prior to assessment. Our findings provide strong evidence for symbiosis between host and microbiomes but we cannot reveal whether the interactions are adaptive, nor whether they are caused by genetic or ecological factors.

## Introduction

Tools in molecular biology allow for assessment of molecular phenotypes such as the transcriptome, the proteome, and the metabolome as well as for sequencing not only host organisms but also the myriad of microorganisms that coexist within these hosts^1,2^. The simultaneous study of hosts and their microorganisms is a fast developing and novel research field that opens for investigating the importance of microbial diversity, how the abundance and distribution of different microbes affect the host’s fitness and how evolutionary and ecological forces shape the symbiosis between host and its microbes^3–5^.

Studies on a variety of species have documented that the microbiome has strong impact on host fitness components including lifespan, fecundity, immune responses, disease resistance, growth, and environmental stress tolerance^6–13^. It has also been shown that the host can exert some control of the microbial composition, e.g. through nutrient availability by diet choice or host metabolism^14–16^, by immune factors^13^, or mechanical control such as gut peristalsis^17^. Further, high abundance of certain species of *Lactobacillus* or *Acetobacter* in nutrient poor environments can critically increase *Drosophila melanogaster* larval growth so that rate of growth and final weight reach those normally observed on high-protein diets^18,19^, suggesting a rescue effect offered by these microbes. Thus, interactions between the host genotype and the microbiota can have strong consequences for the host^20^.

The growing realization that interactions between microbes and their hosts shape many aspects of life is fundamentally changing the way we think about many fields of biology including evolutionary and conservation genetics, disease etiology, and adaptation to changing environmental conditions^5,21–24^. The ‘holobiont’ concept describes the idea that eukaryote individuals do not act as autonomous units, but rather as networks consisting of the host and all its associated microorganisms. Their collective genomes, the ‘hologenome’, may form a single unit of selection and it has been proposed that this hologenome can evolve^25,26^. For instance, it has been shown that selection for increased cold tolerance in the blue tilapia (*Oreochromis aureus*) led to significant alteration of the microbial composition and modulated the microbes’ response to temperature stress in this tropical fish^27^. Likewise, a study investigating symbiosis between the aphid *Acyrthosiphon pisum* and the endosymbiont *Buchnera aphidicola*, has revealed how a single nucleotide deletion in the bacterium affects the heat-shock transcriptional promoter for ibpA, encoding a small heat-shock protein and that this mutation governs thermal tolerance of the aphid hosts^28^. There are examples providing evidence for co-evolution between host and microorganisms e.g. from coral associated bacteria^29^ but symbiosis can also arise from stochastic and / or deterministic ecological forces and the bases for host—microorganism associations are typically lacking in microbiome studies. The importance of ecological factors in shaping the microbiome has for example been revealed in *D. melanogaster* where it was shown that the microbiome can respond to thermal acclimation, and subsequently contribute to improve the host’s survival to more extreme thermal conditions^12^. Studies like these suggest that the microbiome has strong importance for the host’s ability to cope with variable and periodically stressful environmental conditions but they do not reveal whether host and microbiomes evolve in concert.

Genomic studies aiming at pinpointing signatures of selection often report little evidence for selection at the genomic or molecular phenotypic level despite strong directional selection and apparent phenotypic responses to selection^30–32^. One reason for this is that most variation in quantitative genetic traits is influenced by a very large number of genes each contributing with a small effect on the trait in question^33^. Identifying these genes, transcripts or proteins of small effect is a challenge from a statistical point of view^34^. However, an overlooked reason for the apparent lack of strong signatures of selection may be that only parts of the hologenome, the host genome, is traditionally being investigated in these studies. Changes in composition, abundance, and evolution of the myriad of microbial genomes impacted by selection may explain a significant proportion of the response in host phenotypes not captured by analysis of changes in host genomes and this challenge existing evolutionary models^35^. It has been suggested that the microbial composition is influenced by host genotype in *D. melanogaster* and that host genetic control of the microbiome contributes to the genotype–phenotype relationship^36^. This idea led us to investigate the microbial composition in *D. melanogaster* from an artificial selection experiment where replicated lines were selected for increased heat, cold, desiccation and starvation tolerance or longevity, respectively^37^. Flies from these lines were reared at common garden conditions for two generations prior to sampling individuals to be used for sequencing of their microbiomes. This material allowed us to test the hypothesis that directional selection for numerous fitness components in *D. melanogaster*, leads to selection-regime-specific changes in microbial composition. We found increased microbial diversity in selection regimes compared to the unselected control regime and marked differences in microbial composition between many of the selection regimes suggesting that the microbiome has changed alongside with the host. These results provide qualitatively support for phylosymbiosis^4^ but further studies are needed to evaluate whether different microbial composition and diversity across selection regimes are adaptive, whether they are important for explaining phenotypic characteristics of the host, and if they are a result of co-evolution or ecological factors.

## Material and methods

### Description of selection regimes and original experimental setup

The flies used in the current study originated from a large environmental-stress and life-history trait selection experiment described previously^37^. In short, initially a mass-bred laboratory population of *D. melanogaster* was established based on wild caught individuals and maintained at 25 °C in 12-h light–dark cycles, on a medium composed of yeast (60 g/L water), sucrose (40 g/L water), oatmeal (30 g/L water), and agar (16 g/L water) mixed with tap water. Following autoclaving, nipagin (12 mL/L water) and acetic acid (1.2 mL/L water) were added. Six artificial selection regimes were established together with one unselected control (UC) regime. For each selection regime and the control regime five independent replicated lines were generated. In each selection regime selection for one of the following traits was performed: increased desiccation resistance (DS), increased longevity (LS), increased cold-shock resistance (CS), increased heat-shock resistance (HS), increased heat knockdown resistance (KS) and increased starvation resistance (SS). Flies from the UC regime were maintained without deliberate selection, and after emergence flies were aged for 5 days and then placed in a new set of bottles to propagate the next generation. In the DS selection regime 5 days old flies were transferred to empty vials, which were put in an exsiccator containing silica gel (Sorbsil^®^ C, Oker-Chemie GmbH). The exsiccator was placed at room temperature (22–24 °C) and humidity was close to zero after 2 h. Exposure time to desiccation stress was increased from 12 to 20 h during the course of the experiment. The surviving flies were used to establish the next generation. In the LS regime flies were placed in vials with the standard medium immediately after emergence. Every second day they were transferred to fresh food vials until the desired mortality was reached. This period was 4 weeks at the beginning of the experiment and was later increased to 6 weeks. Individuals that were alive after > 4 weeks were used to establish the next generation. In the CS regime we exposed 2 d old flies to 11 °C for 5 days. These cold acclimated flies were subsequently exposed to 0.5 °C for 27 h. The exposure time was gradually increased to 50 h following changes in cold resistance of the selected lines. After cold exposure flies were allowed to recover for 24 h and the survivors were used to establish the next generation. In the HS regime 5 d old flies were exposed to 36 °C for 30 min followed by a return to 25 °C for 24 h. After 24 h of recovery from the hardening treatment (at 36 °C) flies were exposed to a heat shock for 38.0 °C for 1 h. The exposure temperature was gradually increased to 38.5 °C as the flies improved their resistance in response to selection. The surviving flies generated the next generation. In the KS regime 5 day old flies were exposed to a heat knock down treatment^38,39^ at 40 °C. When the flies succumbed due to heat stress they reached a collecting vial, which was replaced every 30 s. Each individual fly was assigned a knockdown time according to when it fell into the collecting vial. Flies with longer knockdown time were mixed and transferred to culture bottles to start a new generation. The SS regime consisted of exposing flies to pure agar medium (2% of agar) to provide moisture and avoid desiccation while starving. The time needed to reach the desired mortality was gradually increased from 35 to 60 h across generations with selection. The survivors founded the next generation. The selection was applied every second generation to allow for recovery and to reduce transgenerational effects^40,41^. For further details regarding selection procedures and maintenance we refer to Bubliy and Loeschcke^37^.

**Figure 1:**
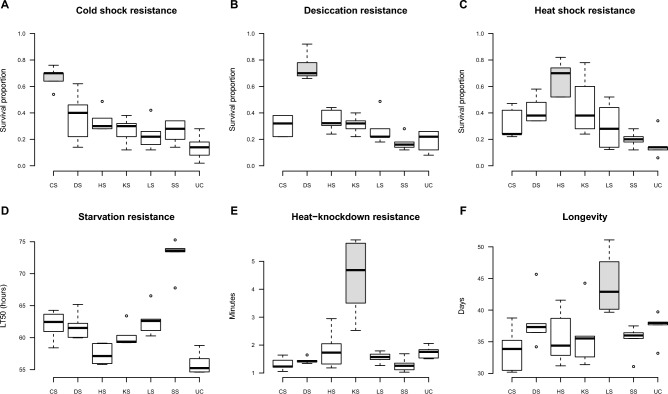
Boxplots showing cold-shock resistance (**A**), desiccation resistance (**B**), heat-shock resistance (**C**), all measured as survival proportions, and starvation resistance (**D**), heat-knockdown resistance (**E**) and longevity (**F**) measured as respectively LT50 in hours, survival after exposure to heat stress in minutes, and survival duration in days. Flies from five replicate lines were investigated per selection regime (selection for increased cold resistance (CS), desiccation resistance (DS), heat shock resistance (HS), starvation resistance (SS), heat-knock down resistance (KS), and longevity (LS), respectively) for all traits, including flies from five unselected control lines (UC). Selection for increased stress resistance or longevity was performed every other generation and populations were selected for 11–27 generations (depending on the selection regime) prior to testing using a common garden design where flies from all regimes were reared at benign conditions (like those at which UC flies were reared across all generations) for two generations before being tested (for details see Bubliy and Loeschcke (2005)^37^ and description in “Material and methods” below). Grey colored boxes in each panel are used to indicate how the lines that are directionally selected for a given trait, e.g. cold shock resistance, perform when tested for that specific trait. All data are from Bubliy and Loeschcke^37^.

The initial phenotypic assessment was performed after 21 generations of selection (45 generations in total), except for LS and SS which were selected for 11 and 26 generations prior to assessment, respectively^37^ (Fig. 1). Flies from each of the selection regimes (and the unselected controls) have previously been characterized at the genomic level^31^, and on a range of different molecular phenotypic levels using transcriptomic, proteomic, and metabolomic techniques^42–45^. The flies used in the current study were collected after 32 generations of selection (i.e., 67 generations of maintenance) for the HS, CS, KS, DS and UC regimes, after 37 generations of selection for the SS regime (i.e., 81 generations of maintenance), and after 14 generations of selection for the LS regime (i.e., 31 generations of maintenance). The different numbers of generations with selection that flies have been through at the times of collection is due to experimental procedures with e.g., longer generation intervals for longevity selection where the long surviving individuals were used as sires and dams to establish the next generation. Phenotypic assessments of selection responses were not performed in the generation where flies were harvested for microbiome analysis and therefore the responsiveness to selection is based on results from the data provided in Bubliy and Loeschcke^37^. Flies used in the omics characterizations^31,42–45^ were harvested at different time points/selection generations and they were exposed to other environmental conditions prior to sampling than flies used in the current study. Therefore, and because we do not have sufficient statistical power, we argue that it is not relevant, nor feasible, to perform analyses associating these data with our microbiome data.

### Microbiome community analysis by 16S rRNA quantification

A total of 20 2–4 days old females were sampled from each of the five replicate lines of each selection and control regime (35 lines in total), snap frozen in liquid nitrogen, and stored at −80 °C for 14 years before performing the microbiome analysis reported here. Flies were not surface sterilized prior to, or post, freezing. Prior to freezing, flies from all lines were reared at benign environmental conditions like those experienced by the UC regime throughout the duration of the experiment, for two generations. Flies were density controlled during these two generations by sampling 40 eggs into five vials with 7 mL medium per line.

DNA extraction and sequencing of 16S rRNA were conducted externally by DNASense ApS (Aalborg, Denmark, https://dnasense.com/). DNA was extracted from pools of 20 female flies (4 from each of the 5 vials) per line using DNeasy Blood and Tissue kit following the manufacturer’s recommendations (Qiagen, Germany). Bacterial 16S V1–V3 rRNA libraries were prepared using a custom protocol based on Caporaso et al.^46^ and amplified using V1-V3 specific primers: [27F] AGAGTTTGATCCTGGCTCAG and [534R] ATTACCGCGGCTGCTGG^47^. The amplicon libraries were purified using a standard protocol for Agencourt Ampure XP Beads (Beckman Coulter, USA) with a bead to sample ratio of 4:5. Purified libraries were pooled in equimolar concentrations and diluted to 6 nM. Samples were paired-end sequenced (2 × 300 bp) on a MiSeq platform (Illumina, USA) using the MiSeq Reagent kit v3 (Illumina, USA) according to standard guidelines.

### Bioinformatic processing and analysis

Demultiplexed fastq files were processed with QIIME2 v2022.2^48^. Forward and reverse primers were removed using the cutadapt plugin v2019.10^49^. To infer amplicon sequence variants (ASVs), trimmed reads were quality filtered, denoised, merged, and PCR chimeras were removed using the DADA2 plugin v2022.2^50^. For quality filtration, paired-end sequences were truncated at 297 and 261 bp for forward and reverse reads, respectively (50-percentile Phred score quality drop below 30 and 28, respectively). DADA2 default parameter settings were applied otherwise. A total of 304 ASVs were detected. ASVs were taxonomically assigned using a 16S V1-V3 specific Naive Bayes classifier^51^. The classifier was trained on 99% similarity clustered 16S rRNA gene sequences extracted from the SILVA v138 reference database^52^ and trimmed to only include the V1-V3 region bound by the 27F/534R primer pair. For phylogenetic inference, ASV sequences were aligned with Mafft v7.310 (Multiple Alignment using Fast Fourier Transform;^53^, via the QIIME2 q2-alignment plugin, including masking of phylogenetically uninformative positions (–p-max-gap-frequency 1, –p-min-conservation 0.4). An unrooted phylogenetic tree was constructed with FastTree v2.1.10^54^, and the tree was rooted by the midpoint of the longest tip-to-tip distance in QIIME2.

Subsequent microbial data analyses were performed with R version 4.1.1^55^. ASVs unassigned at phylum level, ASVs assigned as cyanobacteria, and ASVs assigned as the endosymbiont *Wolbachia* (*Wolbachia* can confound analysis of microbiomes due to extreme high abundances in some lines^56^) were removed (302 ASVs remaining) and remaining ASVs were prevalence filtered and pruned, removing ASVs present in less than two samples and with total abundance less than 0.01% across all samples (206 ASVs remaining, representing 67.76% of detected ASVs). Rarefaction curves were generated with the vegan package v2.5–7^57^ and uneven sampling depth was scaled to minimum read depth (after filtration) by rarefying data to the minimum number of reads across all samples (2873 sequence reads per sample) using the *rarefy_even_depth* function implemented in the phyloseq package v1.38.0^58^. If not stated directly, subsequent microbial composition analyses were conducted using prevalence filtered, pruned, and rarefied ASV count data. Top 10 most abundant bacterial genera across, as well as within, selection regimes were identified and visualized as heatmaps using the ampvis2 package v2.7.11^59^. ASV counts were converted to within-sample relative abundances and for each sample, microbial compositions were visualized at phylum and family level using the microbiome package v1.16^60^.

### Intra-sample complexity

Alpha diversity estimates including the observed number of ASVs (richness) and the Shannon´s diversity index (considering richness and evenness of ASV abundances) were computed with phyloseq. Faith´s phylogenetic diversity (PD) index, a measure of diversity which incorporates phylogenetic diversity between ASVs, was estimated with the picante package v1.8.2^61^. Overall differences in alpha diversity between selection regimes were tested using a Kruskal–Wallis test followed by pairwise comparisons using a Wilcoxon Rank Sum test with Benjamini–Hochberg multiple hypothesis test correction^62^.

### Inter-sample complexity

Differences in microbial community composition between the selection and control regimes were investigated by Principal Coordinate Analysis (PCoA) using weighted UniFrac distances. Weighted UniFrac distances among samples were estimated using phyloseq. PCoA ordination plots were generated with the ggplot2 package v3.3.5^63^. To determine differences in microbial community composition between selection regimes a permutational multivariate analysis of variance (PERMANOVA) was computed for weighted UniFrac distances using the *adonis* function implemented in vegan, applying 999 permutations and default settings otherwise. Homogeneity of group dispersions (variance) was verified using the *betadisper* function implemented in vegan. *P*-values ≤ 0.05 were considered statistically significant. In addition, a hierarchical cluster analysis for weighted UniFrac distances was computed using the *hclust* function implemented in the stats package^55^ applying default settings and visualized in a dendrogram using ggplot2.

### Differential abundance analysis

Differential ASV abundances between selection regimes were determined at genus level for prevalence filtered and pruned (not rarefied) ASV counts present in at least 10% of the samples and collapsed to genus level using a negative binomial generalized linear model approach implemented in the DESeq2 package v1.3.4.^64^. Briefly, ASV counts were normalized applying the variance-stabilizing transformation approach implemented in DESeq2, returning log2 scale transformed data. Size factors for each ASV were estimated applying a median-ratio-method. Dispersions of ASV counts were computed and a negative binomial WaldTest was performed^64,65^. For ASV with zero counts a pseudo-count of one was added^66^. Pairwise comparisons were computed for the following six contrasts: CS vs. UC, DS vs. UC, HS vs. UC, KS vs. UC, LS vs. UC, and SS vs. UC. To correct for multiple hypothesis testing, *P*-values were adjusted using the Benjamini–Hochberg procedure^62^. ASVs were considered differentially abundant when FDR ≤ 0.05. Results were visualized using ggplot2 using R version 4.1.1.

## Results

Illumina 16S rRNA amplicon sequencing returned 1,538,911 raw sequence reads across all 35 samples, ranging from 21,712 to 70,668 reads per sample (mean 43,969). After pre-processing with QIIME2 988,415 sequencing reads remained, representing 64.27% of the raw sequence reads on average. Pre-processed reads detected per sample ranged from 13,723 to 52,185 (mean 28,240). Model based inference of amplicon sequence variants revealed 304 bacterial amplicon sequence variants (ASVs) across all samples, ranging from 12 to 110 unique ASVs detected per sample (mean 58.51). A total of 206 bacterial ASVs passed filtration procedures (per-sample range: 11 to 102 ASVs; mean 54.77 ASVs).

### Microbial composition

**Figure 2:**
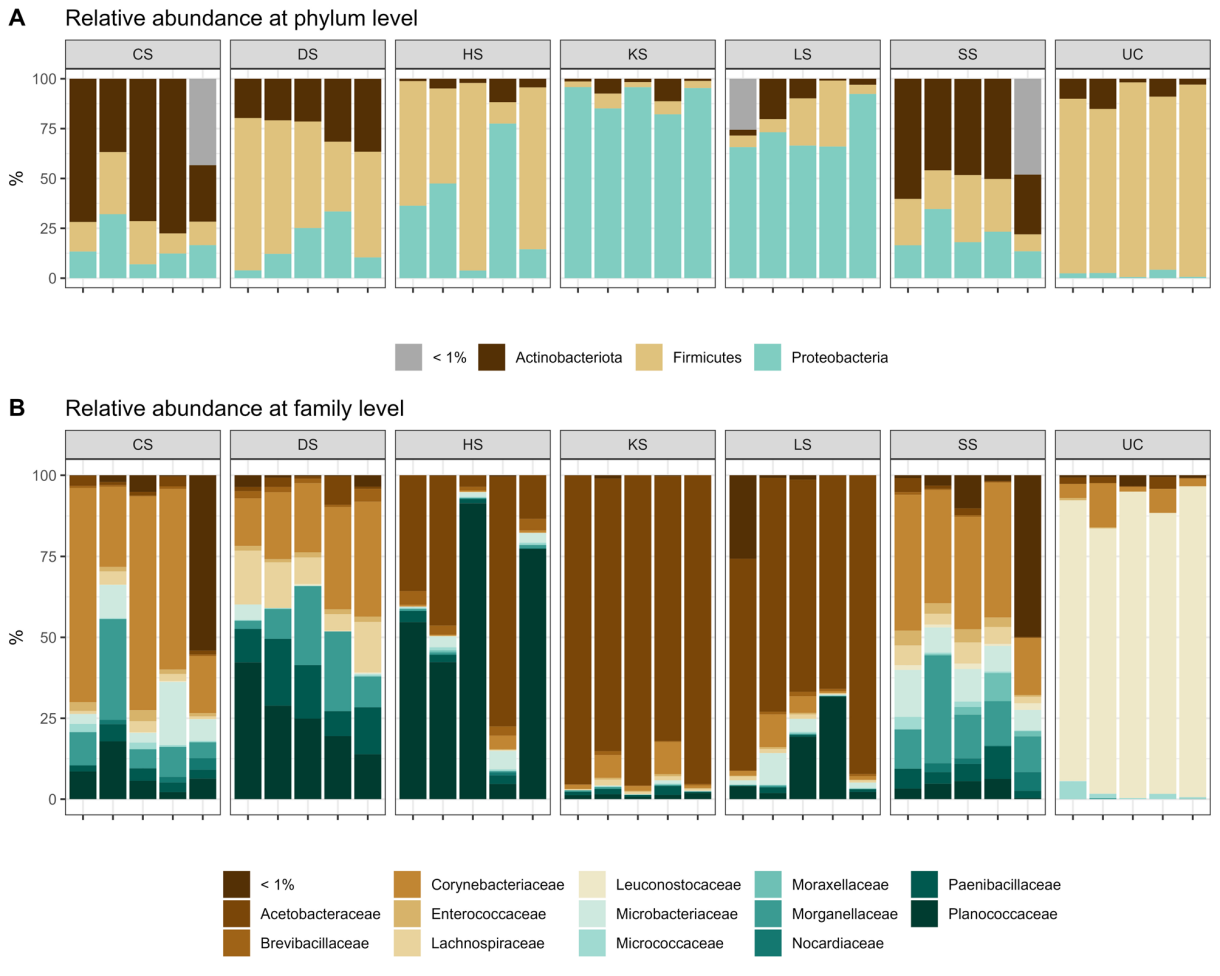
Relative abundance of amplicon sequence variants (ASVs) assigned to the classification level phylum (**A**) and family (**B**) for the six selection regimes (CS cold-shock resistance, DS desiccation resistance, HS heat-shock resistance, KS heat knockdown resistance, LS longevity, SS starvation resistance) and the unselected control regime (UC). Each column within selection regime represents a line.

Detected ASVs were assigned to five phyla: Firmicutes (N = 229; after filtration: N = 157), Actinobacteriota (N = 36; after filtration: N = 26), Proteobacteria (N = 34; after filtration: N = 22), Bacteroidetes (N = 4; after filtration: N = 1), and Cyanobacteria (N = 1; after filtration: N = 0). Relative abundances at phylum and family level are summarized in Fig. 2.

Comparison across selection regimes revealed a change in microbial composition following exposure to artificial selection (Fig. 2). At phylum level (Fig. 2A), samples separated into three groups; samples characterized by high abundance of Proteobacteria (KS, LS), high abundance of Firmicutes (HS, DS, and UC), or high abundance of Actinobacteriota (CS and SS). At family level (Fig. 2B), four microbial profiles were identified: samples with high abundance of (i) *Corynebacteriaceae* (CS, DS, SS), (ii) *Acetobacteraceae* (KS, LS), (iii) *Planococcaceae* (HS), or (iv) *Leuconostocaceae* (UC). Moreover, the most abundant family within each selection regime was sufficient to unambiguously separate selection regimes into the four microbial profiles detected at family level (Fig. S1). Top abundant genera identified included *Acetobacter* representative for KS and LS regimes, *Corynebacterium* representative for CS, DS, and SS, *Lysinibacillus* representative for the HS regime, and *Leuconostoc* representative for the UC regime. Compared to the other detected key genera, *Leuconostoc* was exclusively found in the UC regime.

### Intra-sample complexity

**Figure 3:**
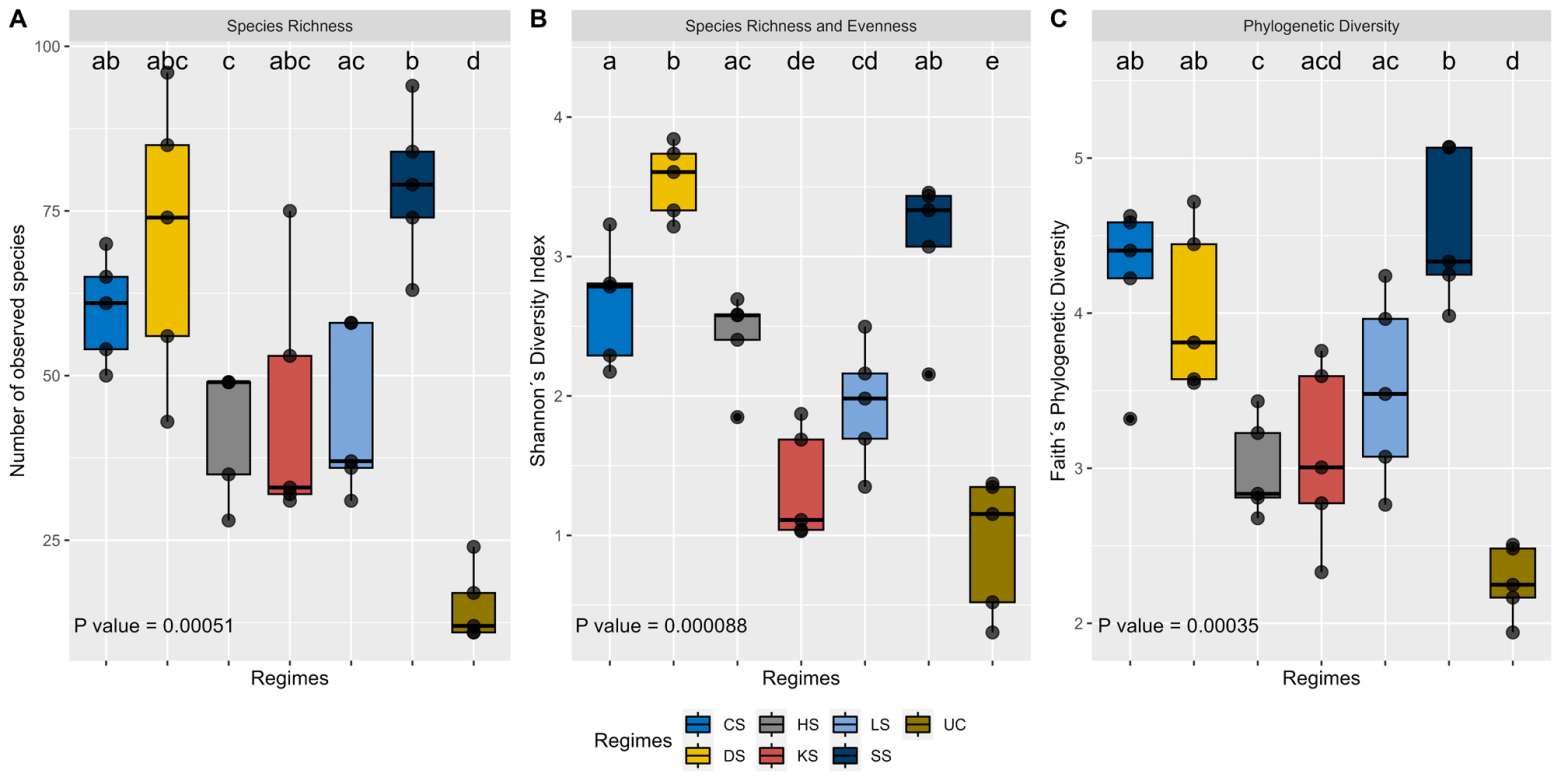
Boxplot for observed (**A**), Shannon’s (**B**) and Faith’s phylogenetic (**C**) alpha-diversity metrics. Letters above each boxplot indicate pairwise statistical differences in α-diversity metrics between regimes (CS cold-shock resistance, DS desiccation resistance, HS heat-shock resistance, KS heat knockdown resistance, LS longevity, SS starvation resistance, UC unselected controls). Global comparisons were conducted using a Kruskal–Wallis test. Pairwise comparisons were conducted using the Wilcoxon Rank Sum test with Benjamini–Hochberg multiple hypothesis test correction. Full statistical reports are found in Supplementary Tables S1–S3.

Within-sample diversity (i.e., alpha diversity) was investigated for prevalence filtered, pruned, and rarefied ASV count data, only including ASVs with an overall abundance > 0.01% (206 ASVs). Global comparison of alpha-diversity estimates between regimes (six selection regimes and the unselected control regime) showed that alpha-diversity differed between them (Fig. 3).

Non-parametric test statistics (Wilcoxon Rank Sum test) were applied for pairwise comparison and showed abundant differences in alpha diversity metrics between pairs of regimes (Supplementary Tables S1–S3). In general, the control regime showed the lowest level of alpha diversity and lower within-sample diversity compared to the selection regimes (Fig. 3, Supplementary Tables S1–S3).

Three different alpha diversity metrics were estimated to account for differences in richness, evenness, and phylogenetic distance. The observed bacterial richness quantifies the total number of ASV within a sample. The UC regime showed significantly less richness compared with the regimes selected for increased stress resistance or longevity (Fig. 3A). The SS regime displayed highest ASV abundances compared with the other selection regimes (Fig. 3A). Shannon’s diversity index accounts for ASV abundance and evenness, such that samples with comparable numbers of ASVs will show a lower diversity measure when one or few ASVs are overrepresented (uneven distribution). Shannon’s diversity index differed significantly between the control and all but the KS regimes (Fig. 3B), suggesting that ASVs detected in the selection regimes were more evenly distributed compared to the control regime. Finally, the Faith’s phylogenetic diversity metric incorporates the phylogenetic distance between ASVs, such that related ASVs increase the measure of phylogenetic diversity less than unrelated ASVs. Like with the observed richness, the control regime had a significant lower estimate of Faith’s phylogenetic diversity metric than any of the selection regimes, and the SS regime displayed the highest diversity metric (Fig. 3C).

### Inter-sample complexity

**Figure 4:**
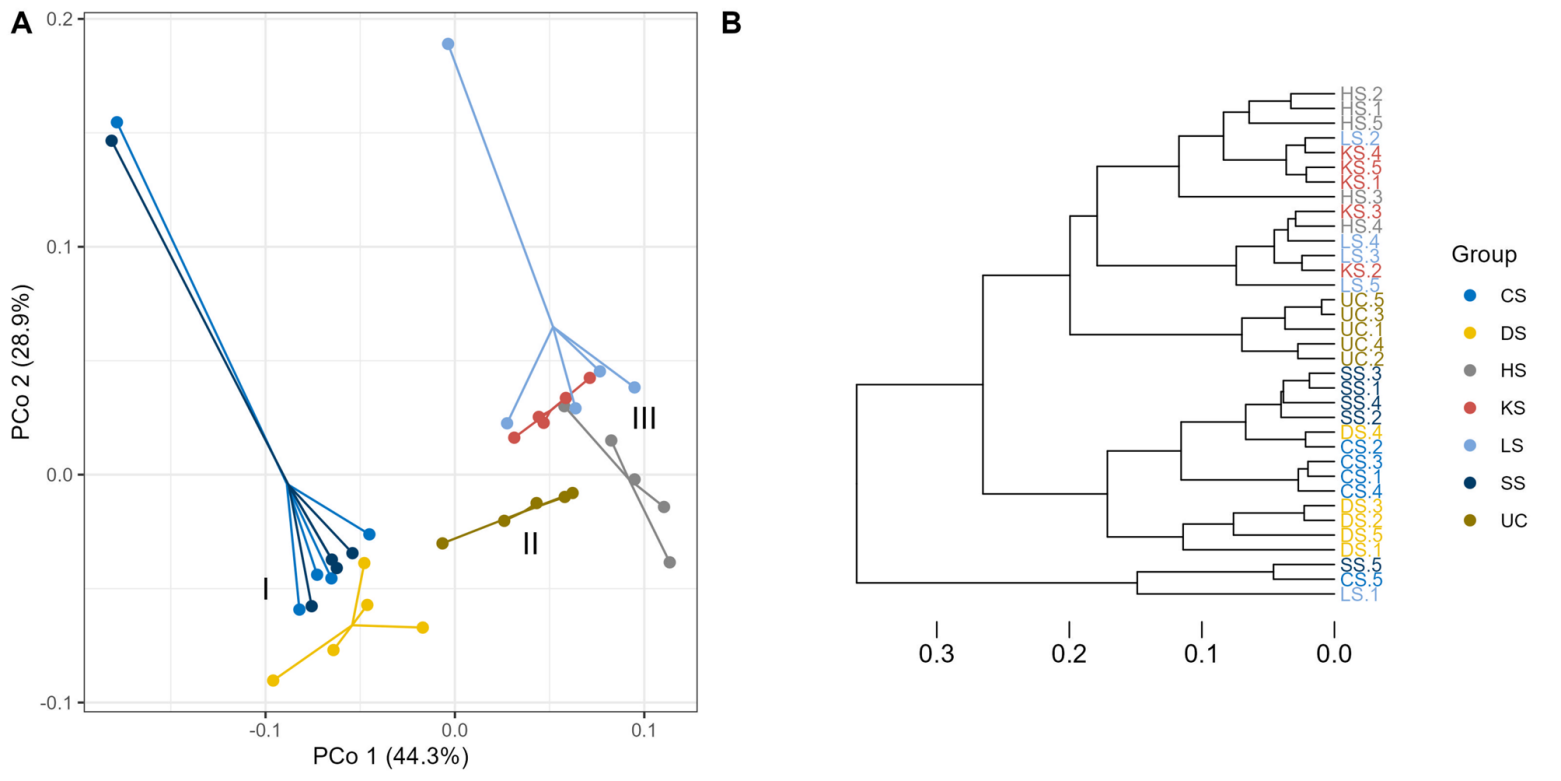
Principal Coordinate (PCo) ordination plot based on weighted UniFrac distances showing PCo 1 and PCo 2, with proportion of variance explained noted (**A**), and cluster analysis (**B**). Roman numbers indicate distinct separate clusters.

Among-sample complexity, i.e., beta diversity, was visualized with Principal Coordinate Analysis (PCoA) ordination plots based on weighted UniFrac distances (Fig. 4). Differences in microbial communities between regimes were detected (Fig. 4). Based on PCo-1 (44.3% of microbial diversity explained) and PCo-2 (28.9% of microbial diversity explained), samples separated into three discrete clusters with CS, DS, and SS regimes forming cluster I, UC regime forming cluster II, and HS, KS and LS regimes forming cluster III. Permutational multivariate analysis of variance (PERMANOVA) revealed that microbial community composition detected in selection regimes clearly differed from the unselected control regime (Table 1). Moreover, microbial compositions of selection regimes belonging to different clusters differed whereas within clusters microbial compositions only differed between CS and DS and SS and DS in cluster I (Table 1) and HS and LS in cluster III (Table 1).

**Table 1:** Permutational multivariate analysis of variance (PERMANOVA) for (A) selection regimes compared with the unselected control regime, (B) pairwise comparison of selection regimes belonging to cluster I (CS, DS, SS), and (C) pairwise comparison of selection regimes belonging to cluster III (HS, KS, LS). PERMANOVA was computed on weighted UniFrac distances.

	Df	Sum sq	R^2^	F value	P value
(A)					
CS vs. UC					
Selection regime	1	0.08	0.58	11.10	0.01
Residuals	8	0.06	0.42		
DS vs. UC					
Selection regime	1	0.07	0.83	37.75	0.005
Residuals	8	0.02	0.17		
HS vs. UC					
Selection regime	1	0.06	0.73	22.176	0.006
Residuals	8	0.02	0.27		
KS vs. UC					
Selection regime	1	0.04	0.83	39.27	0.014
Residuals	8	0.01	0.17		
LS vs. UC					
Selection regime	1	0.06	0.68	17.24	0.013
Residuals	8	0.03	0.32		
SS vs. UC					
Selection regime	1	0.07	0.63	13.41	0.005
Residuals	8	0.04	0.37		
(B)					
CS vs. DS					
Selection regime	1	0.03	0.35	4.33	0.016
Residuals	8	0.06	0.65		
CS vs. SS					
Selection regime	1	0.00	0.05	0.42	0.501
Residuals	8	0.09	0.95		
SS vs. DS					
Selection regime	1	0.03	0.35	4.25	0.008
Residuals	8	0.05	0.65		
(C)					
KS vs. LS					
Selection regime	1	0.00	0.07	0.60	0.804
Residuals	8	0.03	0.93		
HS vs. KS					
Selection regime	1	0.01	0.20	2.02	0.110
Residuals	8	0.02	0.80		
HS vs. LS					
Selection regime	1	0.02	0.33	3.93	0.019
Residuals	8	0.04	0.67		

### Differential abundance

**Figure 5:**
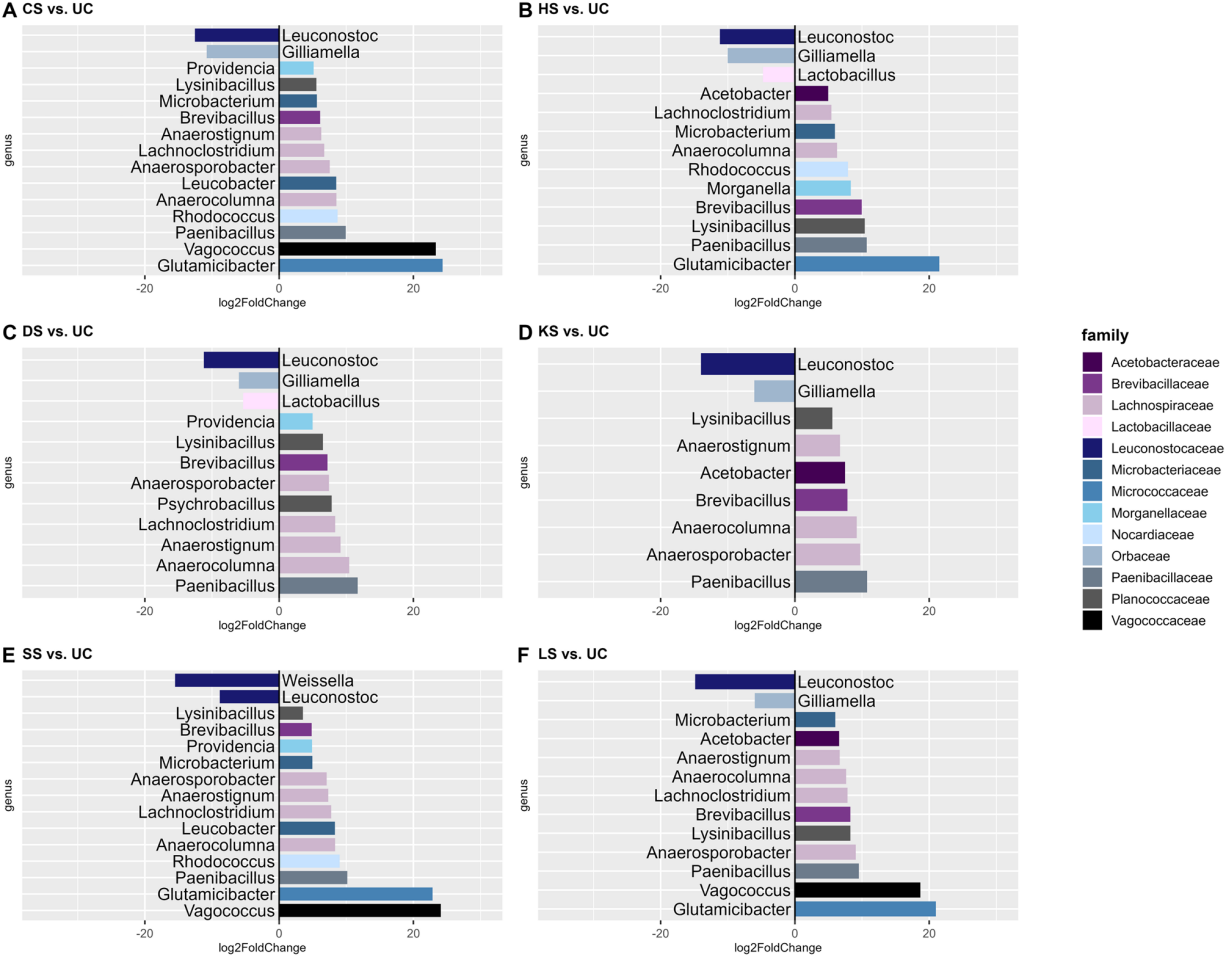
Differential abundance analysis comparing different selection regimes at the genus level. CS cold-shock resistance, DS desiccation resistance, HS heat-shock resistance, KS heat knockdown resistance, LS longevity, SS starvation resistance, UC unselected controls. Color codes applied represents family assignment. Full statistical reports are found in Supplementary Tables S4–S9.

Microbial abundance of the unselected control regimes, agglomerated at genus level, was compared with each of the selection regimes, respectively. Of the 30 assigned genera, a total of 20 genera belonging to 13 different families were significantly affected by at least one selection regime (Fig. 5).

The number of differentially abundant genera ranged from 9 in the KS lines to 15 in the CS and SS lines. The majority of the differentially detected genera (*Acetobacter*, *Anaerocolumna*, *Anaerosporobacter*, *Anaerostignum*, *Brevibacillus*, *Glutamicibacter*, *Lachnoclostridium*, *Leucobacter*, *Lysinibacillus*, *Microbacterium*, *Morganella*, *Paenibacillus*, *Providencia*, *Psychrobacillus*, *Rhodococcus*, *Vagococcus*) showed larger abundances in selection regimes compared to the unselected control regime, with *Anaerocolumna*, *Brevibacillus*, *Lysinibacillus,* and *Paenibacillus* being more abundant across all selection regimes, whereas larger *Morganella* abundance was detected in the HS regime, only (Fig. 5). For the remaining four differentially detected genera (*Gilliamella, Leuconostoc*, *Lactobacillus*, and *Weissella*) abundances were lower in the selection regimes compared to the control regime, with lower *Leuconostoc* abundance detected across all selection regimes, lower *Gilliamella* abundance detected in CS, DS, HS, KS, and LS regimes, lower *Lactobacillus* abundance detected in the DS and HS selection regimes, and lower *Weissella* abundances detected in the SS regime, only. Considering the clusters detected based on beta diversity, *Providencia* was found to be differentially abundant in all selection regimes belonging to cluster I (CS, DS, SS) but in none of the selection regimes belonging to cluster III (HS, KS, and LS). Vice versa, *Acetobacter* was found differentially abundant in all selection regimes belonging to cluster III but in none of the selection regimes belonging to cluster I. Largest increase in abundance (log2 fold-change > 20) was detected for *Glutamicibacter* (CS, HS, LS) and *Vagococcus* (SS) and largest decrease in abundance was observed for *Leuconoctoc* (all selection regimes).

## Discussion

We investigated the microbiomes in replicate unselected control lines, in lines selected for increased longevity, and in lines selected for each of five environmental stress resistance traits, namely increased desiccation resistance (DS), increased cold-shock resistance (CS), increased heat-shock resistance (HS), increased heat knockdown resistance (KS) and increased starvation resistance (SS). Strong genetically based phenotypic responses to selection in these lines have previously been shown (Fig. 1)^31,37,42,44,45,67,68^. Here we found that the microbiome in the selection regimes differed markedly from that of the unselected control regime (Figs. 2 and 3) and that replicate lines within selection regimes had rather similar microbiomes. Lines selected for increased longevity or stress tolerance had increased microbial diversity (Fig. 2) and differential abundance of common *D. melanogaster* microorganisms between some selection regimes (Fig. 5 and supplemental Fig. 1). We found statistical support for three clusters with the CS, SS and DS selection regimes (besides one CS and one SS line) constituting cluster I, UC lines constituting cluster II and KS, HS, and LS selection regimes (besides one LS line) constituting cluster III (Fig. 4). In concordance with previous findings, we show that the microbial community of the host is associated with proxies of fitness components of the host^5–13^.

The flies used for microbiome analysis in our study are not collected in the same generation as the flies used for phenotypic assessment of selection responses reported in Bubliy and Loeschcke^37^ and likewise for flies harvested for omics studies^31,42,44,45,68^. This limits our ability to directly link previous phenotypic and host omics data with the microbiome data. However, we did correlate line phenotypic means reported in Bubliy and Loeschcke^37^ (Fig. 1) with metrics of microbial variation observed in the lines (Figure S2). Results showed consistently high and positive correlations between phenotypic trait values and alpha diversity. Thus, higher microbial diversity is observed in lines with high stress tolerance or longevity in all contrasts involving unselected control lines and selection lines (Figure S2). These strong positive correlations provide no evidence of causation, but they do support other studies revealing host fitness benefits associated with high microbiome diversity^5,35,69^. It has previously been shown that host genetic variation is higher in the *D. melanogaster* selection lines investigated here compared to the unselected control lines^31,68^. Other studies have revealed positive correlations between host genetic variation and microbial diversity^5,70,71^, and it is well-established that also fitness is typically positively correlated with individual and population fitness^72^. Thus, although of correlative nature our results support existing literature proposing that host genetic variation and microbiome diversity are indeed positively associated and that both contribute significantly to host performance. Future studies should address the mechanistic basis and causation explaining this correlation and gut microbiota transfer and rearing e.g. Drosophila under axenic and gnotobiotic conditions will be instrumental in this context^73,74^.

The composition of the host’s microbiome has been linked to variation in the host’s phenotype, such as immune response, metabolism, fitness, and developmental characteristics^75–78^. The microbiome-associated phenotypic mediations can be caused e.g. by the production of metabolites^79^ or the presence of the bacteria per se^80^. For ectotherms in particular, alteration of energy reserves, metabolism, or gene expression caused by alterations in the microbiome may indirectly affect an individual’s environmental stress resistance^81^. In our study we identified three discrete clusters with microbial composition in CS, DS, and SS regimes forming one cluster, the UC regime forming another, and the HS, KS and LS regimes forming a third cluster. Bubliy and Loeschcke^37^ previously showed correlated selection responses across selection regimes, i.e. lines selected for increased heat tolerance were also more tolerant towards other environmental stressors than the unselected control lines. Shared stress responses have previously been observed in Drosophila^44,82^ and the omics studies on the investigated lines reveal evidence for a shared mechanistic basis of the increased stress tolerance in the selection lines^42,44,45^. However as stated elsewhere a quantitative multi-omics analysis is not feasible with our data. What we can state is that we observed abundant changes in the microbial community composition between regimes selected for increased stress resistance or longevity and the unselected control regime. For example, the *Acetobacter* genus*,* which constitutes a group of bacteria known to be related to reduced lipid storage in the host^77,83^ were enriched in HS, KS, and LS selected regimes (cluster III) (Figs. 2 and 5). This aligns with previous findings, indicating that flies acclimated in warmer temperatures exhibit higher *Acetobacter* abundance^12^. Furthermore, it has been reported that gut microbiota may enhance heat tolerance in flies by increasing the expression of heat shock proteins, thereby reducing cellular damage during thermal stress^84–86^. This process is energetically demanding, therefore, the higher abundance of *Acetobacter*, which prioritizes immediate energy utilization over lipid storage, could confer a fitness advantage during heat stress. Conversely, lines with low abundance of *Acetobacter* may have increased lipid storage which is favourable in cold environments^87^. Although no significant differences in *Acetobacter* abundance were found between the unselected control and selection regimes belonging to cluster I (CS, DS, SS), levels were generally low (< 10% relative abundance). The gram positive *Leuconostocaceae* have been identified in wild-caught *D. melanogaster*^88,89^. Here we found that *Leuconostoc* which belongs to the *Leuconostocaceae* family displayed a reduced abundance in all selection regimes compared with the unselected control regime (Fig. 5). Some members of the *Leuconostoc* genus are known to ferment fructose^90^, helping the host with digestion of fruits or other plant materials. Hence, it can be hypothesized that the costs associated with increased stress resistance^91,92^ or longevity^93,94^ are associated with a reduced ability to process food. *Lactobacillus* has been found to be one of the most common genera within the *Drosophila* microbiome^95^, and it has been shown that flies with only *Lactobacillus* microbes can recapitulate the natural microbiota growth-promoting effects^96^. Importantly, chronical stress dramatically changes the microbiota composition and the following metabolomic signature^97^. Here, we found that *Lactobacillus* was less abundant in the regimes selected for increased heat stress and desiccation resistance (Fig. 5).

The few previous studies that have investigated the effects of within species host genetic variation on the microbiota have mainly focused on genetic variability in the host’s ability to control the presence and/or abundance of specific microbes^5,98,99^. Experimental evidence supporting the hologenome hypothesis, i.e., that the host and microbiome genomes evolve in concert and constitute a unit of selection, is however beginning to emerge. For example, Kokou et al.^27^ showed alteration of the microbial composition and microbial response to temperature stress in a tropical fish selected for increased cold resistance, which is in line with the results presented here. In our study we reared *D. melanogaster* selected for different traits under common garden conditions and found noticeable differences in their microbial composition. We observed that the microbiome was generally more diverse in flies selected for increased stress tolerance and longevity and that certain genera were more abundant in some, or all selection regimes compared to the unselected control and vice versa. These findings provide evidence for symbiosis between host and microbiomes which may be caused by co-evolution of host and associated microbiomes or be an ecological consequence of exposure to different environmental conditions prior to harvesting the flies. An experimental evolution study on *D. melanogaster* exposed to a poor nutritional diet have previously shown that the dependency of microbiota for growth and survival is diminished and may be relaxed by the host evolving^100^, which goes against the idea that microbiome composition is evolving in concert with the host. Also, although we attempted rearing all flies in a common garden set up for two generations prior to harvesting we cannot rule out that transgenerational effects of previous stress exposure have an influence on the observed microbial compositions. Also, we do not know whether environmental conditions differ slightly between selection regimes in the common garden set up. Given that the microbiome of *D. melanogaster*, are mainly horizontally acquired^101^, and consist mostly of microbes from the diet and from microbes expelled into the environment^90^ it would have been great to be able to assess microbes in the medium as well. Unfortunately, this was not possible. Environmentally induced immune responses partly govern microbial gut composition^102^ and thus even small differences in rearing conditions can have impacted immunity and thereby the findings in the current study. This is highly relevant in the context of interpretating our results because immune pathways can regulate the microbiota in *D. melanogaster* through antimicrobial peptides^103^.

Our study lags causative interference, and we cannot conclude whether the differences in microbial composition between selection regimes are adaptive and whether they are a consequence of co-evolution or ecological factors. Still, we argue that our findings are both novel and highly important and pave the way for detailed follow-up studies testing hypothesis that can be generated based on our findings. This could involve studies enriching host microbiomes with specific bacteria or with gut content from flies with superior phenotypic characteristic to investigate whether e.g. stress tolerances or longevity can be changed in recipient flies by changing the microbiota.

### Supplementary Information

Supplementary Information.

## Data Availability

Generated 16S sequence data were deposited in FASTQ format in the Sequence Read Archive, National Library of Medicine, National Center for Biotechnology Information, under project number PRJNA1086572. http://www.ncbi.nlm.nih.gov/bioproject/1086572.
