# Supplementary material for: Selection for stress tolerance and longevity in Drosophila melanogaster have strong impacts on microbiome profiles
Source: Sci Rep. 2024 Aug 1;14:17789. doi: 10.1038/s41598-024-68753-5 (PMC11294339; doi:10.1038/s41598-024-68753-5)
Supplement: Supplementary file 1 — Supplementary Information. [file 41598_2024_68753_MOESM1_ESM.pdf]

Supplementary material for

Selection for stress tolerance and longevity in *Drosophila melanogaster* have strong impacts on microbiome profiles

---

The supplementary material contains the following:

Supplementary Figures

Figure S1. Relative abundance (%) of top 10 most abundant amplicon sequence variants (ASVs) assigned to family level.
Figure S2. Scatter plots showing the relationship between gut microbial alpha diversity and phenotypic measures of environmental-stress or life-history traits.

Supplementary Tables

Table S1. Non-parametric pairwise test statistics for observed diversity.
Table S2. Non-parametric pairwise test statistics for Shannon's diversity index.
Table S3. Non-parametric pairwise test statistics for Faith´s phylogenetic diversity index.
Table S4. Differential abundance analysis comparing lines selected for cold-shock resistance (CS) to the unselected control lines (UC) at the genus level.
Table S5. Differential abundance analysis comparing lines selected for desiccation resistance (DS) to the unselected control lines (UC) at the genus level.
Table S6. Differential abundance analysis comparing lines selected for heat-shock resistance (HS) to the unselected control lines (UC) at the genus level.
Table S7. Differential abundance analysis comparing lines selected for heat knockdown resistance (KS) to the unselected control lines (UC) at the genus level.
Table S8. Differential abundance analysis comparing lines selected for longevity (LS) to the unselected control lines (UC) at the genus level.
Table S9. Differential abundance analysis comparing lines selected for starvation resistance (SS) to the unselected control lines (UC) at the genus level.

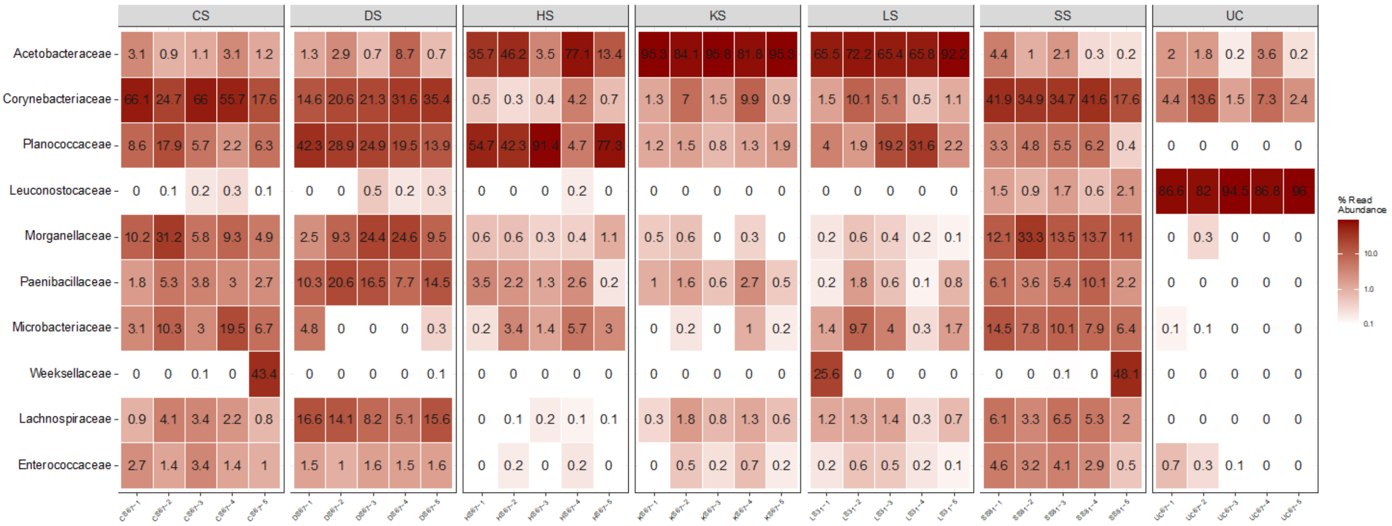

Figure S1. Relative abundance (%) of top 10 most abundant amplicon sequence variants (ASVs) assigned to family level across the six selection regimes (CS: cold-shock resistance, DS: desiccation resistance, HS: heat-shock resistance, KS: heat knockdown resistance, LS: longevity, SS: starvation resistance) and the unselected control lines (UC). Each column within selection type represents the one of the five biological replicates.

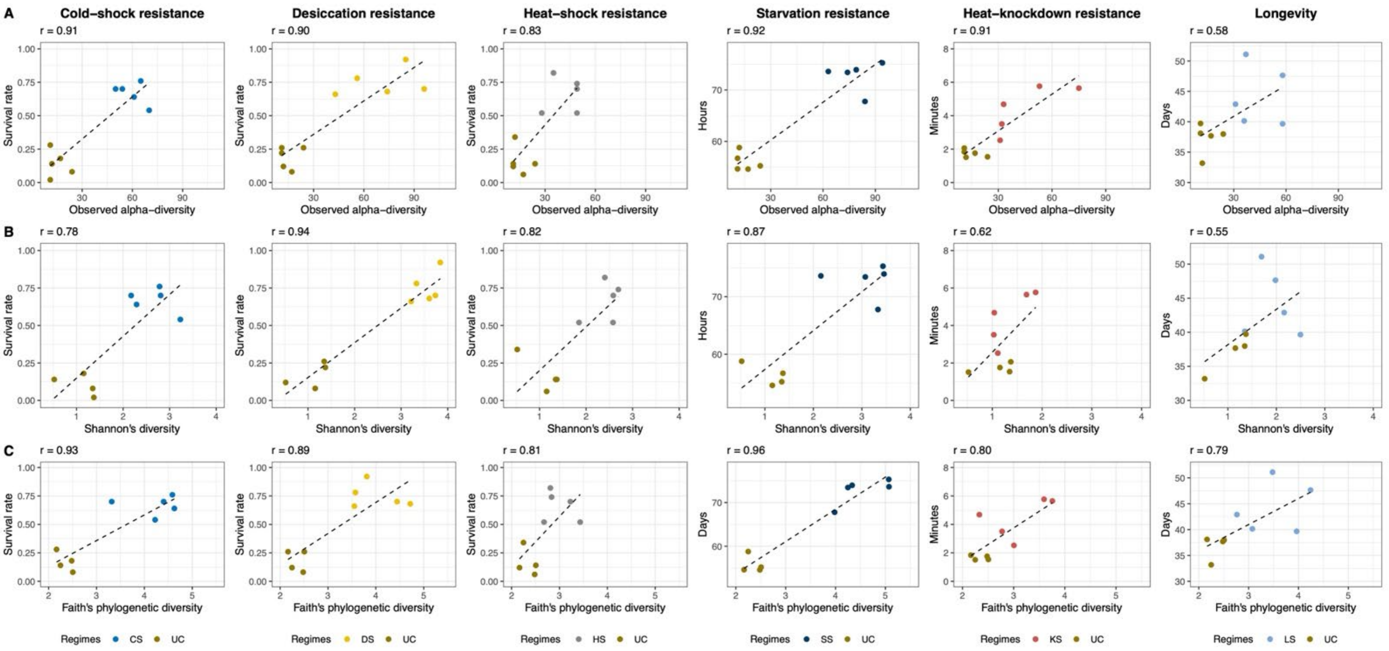

Figure S2. Scatter plots showing the relationship between gut microbial alpha diversity (panel **A**: observed alpha diversity; panel **B**: Shannon´s diversity index; panel **C**: Faith´s phylogenetic diversity index) and phenotypic measures of environmental-stress or life-history traits selected for increased cold-shock resistance, desiccation resistance, heat-shock resistance, all measured as survival proportion, starvation resistance, heat-knockdown resistance and longevity respectively measured as survival duration in days, survival after exposure to heat stress in minutes, and LT50 in hours. Phenotypic traits were recorded for flies from five replicate lines for each of the selection regimes (**CS**: cold resistance; **DS**: desiccation resistance; **HS**: heat shock resistance; **SS**: starvation resistance; **KS**: heat-knock down resistance; **LS**: longevity) and for flies from five unselected control lines (**UC**) and mean phenotypic values were estimated per replication line. Observed, Shannon's, and Faith's Phylogenetic alpha-diversity metrics were estimated per replication line using taxonomy filtered, pruned and rarefied amplicon sequence variant (ASV) data. For each selection regime, alpha diversity of the five UC lines and the five replication lines under selection were plotted against their mean phenotypic trait values and the Pearson´s correlation coefficient (**r**) between alpha diversity and phenotypic trait values were estimated. Colour code applied indicates affiliation with the selection regime. Dashed black lines represents the "line of best fit", minimizing vertical distances between the data points and the line itself, representing the linear trends between alpha diversity and selection responsiveness of phenotypic traits. Selection for increased stress resistance or longevity was performed every other generation and populations were selected for 11-27 generations (depending on the selection regime) using a common garden design. Prior to phenotypic assessment, flies from all regimes were reared at benign conditions (like those at which UC flies were reared across all generations) for two generations (for details see Bubliy and Loeschcke (2005) and description in Material and Methods). Flies used to characterize gut microbial alpha diversity were collected from the same selection experiment but after 14-32 generations of selection depending on the selection regime (i.e., 32 selection events for HS, CS, KS, DS and UC; 37 selection events for SS; 14 events of selection for LS; see Materials and Methods for more details), following benign conditions for two generations prior to sample collection for gut microbiome characterization. Unfortunately, phenotypic assessment of selection responses was not performed in the later generations where flies were harvested for microbiome analysis, hence, correlations between microbial diversity and phenotypic responsiveness to selection could not be investigated within the same individual and should only be considered as proxy.

Table S1. Non-parametric pairwise test statistics (Wilcoxon Rank Sum test with Benjamini–Hochberg multiple hypothesis test correction) for observed α-diversity (species richness measured as number of observed ASVs). Rows in bold indicate statistically significant differences between pairs of selection regimes.

**Comparison**	**$\Delta\alpha$**	**95% confidence interval**		***P* adj**
DS-CS	13	-7.1	33.1	0.52
**HS-CS**	**-12**	**-23.2**	**-0.8**	**0.03**
KS-CS	-28	-46.3	-9.8	0.29
LS-CS	-24	-37.4	-10.6	0.13
SS-CS	18	5,7	30.3	0.06
**UC-CS**	**-49**	**-57.6**	**-40.4**	**0.03**
HS-DS	-25	-45.7	-4.3	0.10
KS-DS	-41	-66.2	-15.8	0.13
LS-DS	-37	-59.0	-15	0.13
SS-DS	5	-16.3	26.3	0.79
**UC-DS**	**-62**	**-81.4**	**-42.6**	**0.03**
KS-HS	-16	-34.9	2.9	1.00
LS-HS	-12	-26.3	2.3	0.78
**SS-HS**	**30**	**16.7**	**43.3**	**0.03**
**UC-HS**	**-37**	**-47.0**	**-27.0**	**0.03**
LS-KS	4	-16.3	24.3	0.79
SS-KS	46	26.4	65.6	0.06
**UC-KS**	**-21**	**-38.5**	**-3.5**	**0.03**
**SS-LS**	**42**	**26.8**	**57.2**	**0.03**
**UC-LS**	**-25**	**-37.4**	**-12.6**	**0.03**
**UC-SS**	**-67**	**-78.2**	**-55.8**	**0.03**

$\Delta\alpha$ difference α-diversity between pairs of selection regimes.

Table S2. Non-parametric pairwise test statistics (Wilcoxon Rank Sum test with Benjamini–Hochberg multiple hypothesis test correction) for Shannon's α-diversity index. Rows in bold indicate statistically significant differences between pairs of selection regimes.

Comparison	Δα	95% confidence interval		*P* adj
**DS-CS**	**0.8**	**0.4**	**1.3**	**0.03**
HS-CS	-0.2	-0.7	0.3	0.44
**KS-CS**	**-1.7**	**-2.2**	**-1.2**	**0.02**
**LS-CS**	**-0.8**	**-1.3**	**-0.3**	**0.05**
SS-CS	0.6	-0.1	1.2	0.25
**UC-CS**	**-1.6**	**-2.2**	**-1.1**	**0.02**
**HS-DS**	**-1.0**	**-1.4**	**-0.7**	**0.02**
**KS-DS**	**-2.5**	**-2.9**	**-2.1**	**0.02**
**LS-DS**	**-1.6**	**-2.1**	**-1.2**	**0.02**
SS-DS	-0.3	-0.8	0.3	0.25
**UC-DS**	**-2.5**	**-2.9**	**-2.0**	**0.02**
**KS-HS**	**-1.5**	**-1.9**	**-1.0**	**0.03**
LS-HS	-0.6	-1.1	-0.1	0.12
SS-HS	0.8	0.2	1.3	0.12
**UC-HS**	**-1.4**	**-2.0**	**-0.9**	**0.02**
LS-KS	0.9	0.4	1.4	0.08
**SS-KS**	**2.2**	**1.6**	**2.8**	**0.02**
UC-KS	0.0	-0.5	0.6	0.55
**SS-LS**	**1.4**	**-2.9**	**2.0**	**0.05**
**UC-LS**	**-0.8**	**-1.4**	**-0.3**	**0.03**
**UC-SS**	**-2.2**	**-2.8**	**-1.5**	**0.02**

$\Delta\alpha$ difference α-diversity between pairs of selection regimes.

Table S3. Parametric pairwise test statistics (Wilcoxon Rank Sum test with Benjamini–Hochberg multiple hypothesis test correction) for Faith´s phylogenetic diversity. Rows in bold indicate statistically significant differences between pairs of selection regimes.

**Comparison**	**Δα**	**95% confidence interval**		***P* adj**
DS-CS	-0.6	-1.0	-0.2	0.84
**HS-CS**	**-1.6**	**-2.1**	**-1.1**	**0.03**
KS-CS	-1.4	-1.9	-0.9	0.06
LS-CS	-0.9	-1.2	-0.4	0.14
SS-CS	-0.1	-0.7	0.5	0.76
**UC-CS**	**-2.2**	**-2.7**	**-1.6**	**0.02**
**HS-DS**	**-1.0**	**-1.4**	**-0.6**	**0.02**
KS-DS	-0.8	-1.2	-0.4	0.14
LS-DS	-0.3	-0.8	0.1	0.28
SS-DS	0.5	0.0	1.1	0.28
**UC-DS**	**-1.6**	**-2.1**	**-1.1**	**0.02**
KS-HS	0.2	-0.3	0.6	0.84
LS-HS	0.6	0.2	1.1	0.28
**SS-HS**	**1.5**	**0.9**	**2.1**	**0.02**
UC-HS	-0.6	-1.1	-0.1	0.02
LS-KS	0.5	-0.1	1.0	0.49
**SS-KS**	**1.3**	**0.7**	**1.9**	**0.02**
UC-KS	-0.8	-1.3	-0.2	0.06
**SS-LS**	**0.9**	**0.2**	**1.5**	**0.03**
**UC-LS**	**-1.2**	**-1.8**	**-0.7**	**0.02**
**UC-SS**	**-2.1**	**-2.7**	**-1.4**	**0.02**

$\Delta\alpha$ difference α-diversity between pairs of selection regimes.

Table S4. Differential abundance analysis comparing lines selected for cold-shock resistance (CS) to the unselected control lines (UC) at the genus level. Taxonomies were agglomerated to genus level prior to analysis. ASVs are ordered according to adjusted P-values (*P* adj) from lowest to highest. ASVs were considered differentially abundant for adjusted P-values ≤ 0.01 and an FDR cut-off of 5%. Significance threshold (*P* adj = 0.01) is marked by a dashed line.

ASV ID	baseMean	log2FC	*P* adj	Domain	Phylum	Class	Order	Family	Genus
abc8f555ea0b005c9a4085a8343a3f99	326.35	9.93	1.52E-20	Bacteria	Firmicutes	Bacilli	Paenibacillales	Paenibacillaceae	Paenibacillus
cd416ce5bf4afa614154fc0a45feb5a8	20.90	24.35	3.04E-19	Bacteria	Actinobacteriota	Actinobacteria	Micrococcales	Micrococcaceae	Glutamicibacter
810bf4f2547219682f5ab14a09ca3599	4949.58	-12.55	1.83E-18	Bacteria	Firmicutes	Bacilli	Lactobacillales	Leuconostocaceae	Leuconostoc
19492e6d689a95489805fe62a81b5ecd	194.34	6.11	2.01E-10	Bacteria	Firmicutes	Bacilli	Brevibacillales	Brevibacillaceae	Brevibacillus
33b3b41ddb67bb49f482abc6126da323	65.78	7.54	8.37E-10	Bacteria	Firmicutes	Clostridia	Lachnospirales	Lachnospiraceae	Anaerosporobacter
ebc7527f35924613e64e071623af1abb	105.76	8.53	2.19E-09	Bacteria	Firmicutes	Clostridia	Lachnospirales	Lachnospiraceae	Anaerocolumna
c37413af5ea0a7254284c92b8bf54ae7	437.97	5.14	8.92E-07	Bacteria	Proteobacteria	Gammaproteobacteria	Enterobacterales	Morganellaceae	Providencia
d1e46966f512a55ca12a01802729f4ef	43.43	-10.80	1.17E-06	Bacteria	Proteobacteria	Gammaproteobacteria	Orbales	Orbaceae	Gilliamella
148a7bc577920720430585105 30ea32d	3855.11	5.55	6.76E-06	Bacteria	Firmicutes	Bacilli	Bacillales	Planococcaceae	Lysinibacillus
05f5c262eb96bf78b6ab2750fdddc0c4	33.94	6.72	2.46E-05	Bacteria	Firmicutes	Clostridia	Lachnospirales	Lachnospiraceae	Lachnoclostridium
5634c39a6b4a1475938b6ad7a4113445	23.37	23.34	4.22E-05	Bacteria	Firmicutes	Bacilli	Lactobacillales	Vagococcaceae	Vagococcus
9eadd1514e5fcfa10e723634e35f3485	377.67	5.62	7.02E-05	Bacteria	Actinobacteriota	Actinobacteria	Micrococcales	Microbacteriaceae	Microbacterium
c2b73b7534124edd282fa67a545e048b	44.47	8.71	0.00024	Bacteria	Actinobacteriota	Actinobacteria	Corynebacteriales	Nocardiaceae	Rhodococcus
7406188bce39180c4aa194bfb387935e	37.30	6.27	0.00024	Bacteria	Firmicutes	Clostridia	Lachnospirales	Lachnospiraceae	Anaerostignum
c63d552898d6b61e6ef07797210d7aca	25.79	8.51	0.00039	Bacteria	Actinobacteriota	Actinobacteria	Micrococcales	Microbacteriaceae	Leucobacter
19019fa034a9856e8d6365d34574c8fd	544.72	-14.60	0.011	Bacteria	Firmicutes	Bacilli	Lactobacillales	Leuconostocaceae	Weissella
64bfc35722d08fe4ccf6b5665b9d1769	15.77	6.48	0.015	Bacteria	Proteobacteria	Gammaproteobacteria	Enterobacterales	Morganellaceae	Morganella
ffaedab25be5e2be043b2355be6644cf	17.78	6.57	0.032	Bacteria	Firmicutes	Clostridia	Clostridiales	Clostridiaceae	Clostridium_sensu_stricto_1
4e817d860267844d0bb67bb3ed49522c	25.82	-3.00	0.068841952	Bacteria	Firmicutes	Bacilli	Lactobacillales	Lactobacillaceae	Lactobacillus
7fda73c8ad009ec246fc6a5bf669203e	1638.56	1.41	0.079773355	Bacteria	Actinobacteriota	Actinobacteria	Corynebacteriales	Corynebacteriaceae	Corynebacterium
0ff98df849b8fae49800a796dadc506c	93.90	1.64	0.096340551	Bacteria	Firmicutes	Bacilli	Lactobacillales	Enterococcaceae	Enterococcus
7d6f288bd50c0431d8fe542c02c1a0d2	14.62	2.96	0.225500311	Bacteria	Firmicutes	Bacilli	Bacillales	Planococcaceae	Psychrobacillus
f8a687d17d5bcd547799c35ad359bf70	11655.70	-0.54	0.493572561	Bacteria	Proteobacteria	Alphaproteobacteria	Acetobacterales	Acetobacteraceae	Acetobacter

baseMean: Mean abundance of the normalized count values over all samples included in the comparison; log2FC: log2-fold change represents the effect size estimate reported on a logarithmic scale to base 2, indicating how much the ASV abundance has changed due to selection for cold-shock resistance in comparison to the control lines; *P* adj: adjusted P-values using the Benjamini-Hochberg procedure.

Table S5. Differential abundance analysis comparing lines selected for desiccation resistance (DS) to the unselected control lines (UC) at the genus level. Taxonomies were agglomerated to genus level prior to analysis. ASVs are ordered according to adjusted P-values (*P* adj) from lowest to highest. ASVs were considered differentially abundant for adjusted P-values ≤ 0.01 and an FDR cut-off of 5%. Significance threshold (*P* adj = 0.01) is marked by a dashed line.

ASV ID	baseMean	log2FC	*P* adj	Domain	Phylum	Class	Order	Family	Genus
abc8f555ea0b005c9a4085a8343a3f99	326.35	11.70	2.24E-28	Bacteria	Firmicutes	Bacilli	Paenibacillales	Paenibacillaceae	Paenibacillus
810bf4f2547219682f5ab14a09ca3599	4949.58	-11.22	6.81E-15	Bacteria	Firmicutes	Bacilli	Lactobacillales	Leuconostocaceae	Leuconostoc
19492e6d689a95489805fe62a81b5ecd	194.34	7.20	4.78E-14	Bacteria	Firmicutes	Bacilli	Brevibacillales	Brevibacillaceae	Brevibacillus
ebc7527f35924613e64e071623af1abb	105.76	10.44	2.28E-13	Bacteria	Firmicutes	Clostridia	Lachnospirales	Lachnospiraceae	Anaerocolumna
33b3b41ddb67bb49f482abc6126da323	65.78	7.43	2.01E-09	Bacteria	Firmicutes	Clostridia	Lachnospirales	Lachnospiraceae	Anaerosporobacter
148a7bc57792072043058510530ea32d	3855.11	6.53	1.36E-07	Bacteria	Firmicutes	Bacilli	Bacillales	Planococcaceae	Lysinibacillus
7406188bce39180c4aa194bfb387935e	37.30	9.15	1.36E-07	Bacteria	Firmicutes	Clostridia	Lachnospirales	Lachnospiraceae	Anaerostignum
05f5c262eb96bf78b6ab2750fdddc0c4	33.94	8.36	1.47E-07	Bacteria	Firmicutes	Clostridia	Lachnospirales	Lachnospiraceae	Lachnoclostridium
c37413af5ea0a7254284c92b8bf54ae7	437.97	4.99	1.91E-06	Bacteria	Proteobacteria	Gammaproteobacteria	Enterobacterales	Morganellaceae	Providencia
7d6f288bd50c0431d8fe542c02c1a0d2	14.62	7.83	2.39E-03	Bacteria	Firmicutes	Bacilli	Bacillales	Planococcaceae	Psychrobacillus
4e817d860267844d0bb67bb3ed49522c	25.82	-5.36	2.39E-03	Bacteria	Firmicutes	Bacilli	Lactobacillales	Lactobacillaceae	Lactobacillus
d1e46966f512a55ca12a01802729f4ef	43.43	-5.99	5.24E-03	Bacteria	Proteobacteria	Gammaproteobacteria	Orbales	Orbaceae	Gilliamella
19019fa034a9856e8d6365d34574c8fd	544.72	-14.51	1.79E-02	Bacteria	Firmicutes	Bacilli	Lactobacillales	Leuconostocaceae	Weissella
ffaedab25be5e2be043b2355be6644cf	17.78	5.56	1.21E-01	Bacteria	Firmicutes	Clostridia	Clostridiales	Clostridiaceae	Clostridium_sensu_stricto_1
9eadd1514e5fcfa10e723634e35f3485	377.67	2.44	1.42E-01	Bacteria	Actinobacteriota	Actinobacteria	Micrococcales	Microbacteriaceae	Microbacterium
b9a736421dc15f263c93ec9f509d5f6b	4.99	6.64	2.65E-01	Bacteria	Firmicutes	Clostridia	Peptostreptococcales-Tissierellales	Peptostreptococcaceae	Clostridioides
016b118f0377208f78e5694abe0d59b6	4.40	6.65	3.82E-01	Bacteria	Firmicutes	Clostridia	Oscillospirales	Ruminococcaceae	Incertae_Sedis
43f9d96deeccbd4b7c28e80612ec05ea	9.56	-2.99	4.76E-01	Bacteria	Actinobacteriota	Actinobacteria	Micrococcales	Brevibacteriaceae	Brevibacterium
f8a687d17d5bcd547799c35ad359bf70	11655.70	-0.80	4.76E-01	Bacteria	Proteobacteria	Alphaproteobacteria	Acetobacterales	Acetobacteraceae	Acetobacter
0ff98df849b8fae49800a796dadc506c	93.90	0.50	8.72E-01	Bacteria	Firmicutes	Bacilli	Lactobacillales	Enterococcaceae	Enterococcus
c2b73b7534124edd282fa67a545e048b	44.47	0.00	1.00E+00	Bacteria	Actinobacteriota	Actinobacteria	Corynebacteriales	Nocardiaceae	Rhodococcus
cd416ce5bf4afa614154fc0a45feb5a8	20.90	0.00	1.00E+00	Bacteria	Actinobacteriota	Actinobacteria	Micrococcales	Micrococcaceae	Glutamicibacter
c63d552898d6b61e6ef07797210d7aca	25.79	0.00	1.00E+00	Bacteria	Actinobacteriota	Actinobacteria	Micrococcales	Microbacteriaceae	Leucobacter
7fda73c8ad009ec246fc6a5bf669203e	1638.56	-0.22	1.00E+00	Bacteria	Actinobacteriota	Actinobacteria	Corynebacteriales	Corynebacteriaceae	Corynebacterium
64bfc35722d08fe4ccf6b5665b9d1769	15.77	0.00	1.00E+00	Bacteria	Proteobacteria	Gammaproteobacteria	Enterobacterales	Morganellaceae	Morganella
515ecf421a951bc274a7daaa791dc894	5.27	0.00	1.00E+00	Bacteria	Proteobacteria	Alphaproteobacteria	Rhodobacterales	Rhodobacteraceae	Paracoccus
f8d46ee1054ee9224a7e622fd2557cf4	7.49	0.00	1.00E+00	Bacteria	Firmicutes	Bacilli	Paenibacillales	Paenibacillaceae	Cohnella
5634c39a6b4a1475938b6ad7a4113445	23.37	0.00	1.00E+00	Bacteria	Firmicutes	Bacilli	Lactobacillales	Vagococcaceae	Vagococcus
2d5564533c7c3ef0e1f984107091c53c	1.05	1.46	1.00E+00	Bacteria	Firmicutes	Bacilli	Bacillales	Bacillaceae	Bacillus

baseMean: Mean abundance of the normalized count values over all samples included in the comparison; log2FC: log2-fold change represents the effect size estimate reported on a logarithmic scale to base 2, indicating how much the ASV abundance has changed due to selection for desiccation resistance in comparison to the control lines; *P* adj: adjusted P-values using the Benjamini-Hochberg procedure.

Table S6. Differential abundance analysis comparing lines selected for heat-shock resistance (HS) to the unselected control lines (UC) at the genus level. Taxonomies were agglomerated to genus level prior to analysis. ASVs are ordered according to adjusted P-values (*P* adj) from lowest to highest. ASVs were considered differentially abundant for adjusted P-values ≤ 0.01 and an FDR cut-off of 5%. Significance threshold (*P* adj = 0.01) is marked by a dashed line.

ASV ID	baseMean	log2FC	*P* adj	Domain	Phylum	Class	Order	Family	Genus
19492e6d689a95489805fe62a81b5ecd	194.34	9.96	5.67E-26	Bacteria	Firmicutes	Bacilli	Brevibacillales	Brevibacillaceae	Brevibacillus
abc8f555ea0b005c9a4085a8343a3f99	326.35	10.70	4.97E-24	Bacteria	Firmicutes	Bacilli	Paenibacillales	Paenibacillaceae	Paenibacillus
148a7bc57792072043058510530ea32d	3855.11	10.40	1.15E-17	Bacteria	Firmicutes	Bacilli	Bacillales	Planococcaceae	Lysinibacillus
cd416ce5bf4afa614154fc0a45feb5a8	20.90	21.51	3.11E-15	Bacteria	Actinobacteriota	Actinobacteria	Micrococcales	Micrococcaceae	Glutamicibacter
810bf4f2547219682f5ab14a09ca3599	4949.58	-11.17	4.63E-15	Bacteria	Firmicutes	Bacilli	Lactobacillales	Leuconostocaceae	Leuconostoc
f8a687d17d5bcd547799c35ad359bf70	11655.70	4.96	1.59E-09	Bacteria	Proteobacteria	Alphaproteobacteria	Acetobacterales	Acetobacteraceae	Acetobacter
d1e46966f512a55ca12a01802729f4ef	43.43	-10.01	9.80E-06	Bacteria	Proteobacteria	Gammaproteobacteria	Orbales	Orbaceae	Gilliamella
ebc7527f35924613e64e071623af1abb	105.76	6.28	2.12E-05	Bacteria	Firmicutes	Clostridia	Lachnospirales	Lachnospiraceae	Anaerocolumna
9eadd1514e5fcfa10e723634e35f3485	377.67	5.96	3.71E-05	Bacteria	Actinobacteriota	Actinobacteria	Micrococcales	Microbacteriaceae	Microbacterium
05f5c262eb96bf78b6ab2750fdddc0c4	33.94	5.44	1.18E-03	Bacteria	Firmicutes	Clostridia	Lachnospirales	Lachnospiraceae	Lachnoclostridium
c2b73b7534124edd282fa67a545e048b	44.47	7.91	1.37E-03	Bacteria	Actinobacteriota	Actinobacteria	Corynebacteriales	Nocardiaceae	Rhodococcus
64bfc35722d08fe4ccf6b5665b9d1769	15.77	8.33	2.56E-03	Bacteria	Proteobacteria	Gammaproteobacteria	Enterobacterales	Morganellaceae	Morganella
4e817d860267844d0bb67bb3ed49522c	25.82	-4.76	6.99E-03	Bacteria	Firmicutes	Bacilli	Lactobacillales	Lactobacillaceae	Lactobacillus
7fda73c8ad009ec246fc6a5bf669203e	1638.56	-2.18	1.02E-02	Bacteria	Actinobacteriota	Actinobacteria	Corynebacteriales	Corynebacteriaceae	Corynebacterium
33b3b41ddb67bb49f482abc6126da323	65.78	3.47	1.18E-02	Bacteria	Firmicutes	Clostridia	Lachnospirales	Lachnospiraceae	Anaerosporobacter
19019fa034a9856e8d6365d34574c8fd	544.72	-13.79	2.05E-02	Bacteria	Firmicutes	Bacilli	Lactobacillales	Leuconostocaceae	Weissella
0ff98df849b8fae49800a796dadc506c	93.90	-2.21	4.21E-02	Bacteria	Firmicutes	Bacilli	Lactobacillales	Enterococcaceae	Enterococcus
f8d46ee1054ee9224a7e622fd2557cf4	7.49	6.83	5.85E-02	Bacteria	Firmicutes	Bacilli	Paenibacillales	Paenibacillaceae	Cohnella
c37413af5ea0a7254284c92b8bf54ae7	437.97	1.91	8.28E-02	Bacteria	Proteobacteria	Gammaproteobacteria	Enterobacterales	Morganellaceae	Providencia
7406188bce39180c4aa194bfb387935e	37.30	3.15	9.73E-02	Bacteria	Firmicutes	Clostridia	Lachnospirales	Lachnospiraceae	Anaerostignum
ffaedab25be5e2be043b2355be6644cf	17.78	4.53	1.67E-01	Bacteria	Firmicutes	Clostridia	Clostridiales	Clostridiaceae	Clostridium_sensu_stricto_1
43f9d96deeccbd4b7c28e80612ec05ea	9.56	1.40	7.93E-01	Bacteria	Actinobacteriota	Actinobacteria	Micrococcales	Brevibacteriaceae	Brevibacterium
7d6f288bd50c0431d8fe542c02c1a0d2	14.62	0.00	1.00E+00	Bacteria	Firmicutes	Bacilli	Bacillales	Planococcaceae	Psychrobacillus
b9a736421dc15f263c93ec9f509d5f6b	4.99	0.00	1.00E+00	Bacteria	Firmicutes	Clostridia	Peptostreptococcales-Tissierellales	Peptostreptococcaceae	Clostridioides
016b118f0377208f78e5694abe0d59b6	4.40	0.00	1.00E+00	Bacteria	Firmicutes	Clostridia	Oscillospirales	Ruminococcaceae	Incertae_Sedis
c63d552898d6b61e6ef07797210d7aca	25.79	0.00	1.00E+00	Bacteria	Actinobacteriota	Actinobacteria	Micrococcales	Microbacteriaceae	Leucobacter
515ecf421a951bc274a7daaa791dc894	5.27	0.00	1.00E+00	Bacteria	Proteobacteria	Alphaproteobacteria	Rhodobacterales	Rhodobacteraceae	Paracoccus
5634c39a6b4a1475938b6ad7a4113445	23.37	0.00	1.00E+00	Bacteria	Firmicutes	Bacilli	Lactobacillales	Vagococcaceae	Vagococcus
19492e6d689a95489805fe62a81b5ecd	194.34	9.96	5.67E-26	Bacteria	Firmicutes	Bacilli	Brevibacillales	Brevibacillaceae	Brevibacillus

baseMean: Mean abundance of the normalized count values over all samples included in the comparison; log2FC: log2-fold change represents the effect size estimate reported on a logarithmic scale to base 2, indicating how much the ASV abundance has changed due to selection for heat-shock resistance in comparison to the control lines; *P* adj: adjusted P-values using the Benjamini-Hochberg procedure.

Table S7. Differential abundance analysis comparing lines selected for heat knockdown resistance (KS) to the unselected control lines (UC) at the genus level. Taxonomies were agglomerated to genus level prior to analysis. ASVs are ordered according to adjusted P-values (*P* adj) from lowest to highest. ASVs were considered differentially abundant for adjusted P-values ≤ 0.01 and an FDR cut-off of 5%. Significance threshold (*P* adj = 0.01) is marked by a dashed line.

ASV ID	baseMean	log2FC	*P* adj	Domain	Phylum	Class	Order	Family	Genus
abc8f555ea0b005c9a4085a8343a3f99	326.35	10.75	5.65E-24	Bacteria	Firmicutes	Bacilli	Paenibacillales	Paenibacillaceae	Paenibacillus
f8a687d17d5bcd547799c35ad359bf70	11655.70	7.47	4.74E-20	Bacteria	Proteobacteria	Alphaproteobacteria	Acetobacterales	Acetobacteraceae	Acetobacter
810bf4f2547219682f5ab14a09ca3599	4949.58	-14.00	5.33E-20	Bacteria	Firmicutes	Bacilli	Lactobacillales	Leuconostocaceae	Leuconostoc
19492e6d689a95489805fe62a81b5ecd	194.34	7.83	1.27E-16	Bacteria	Firmicutes	Bacilli	Brevibacillales	Brevibacillaceae	Brevibacillus
33b3b41ddb67bb49f482abc6126da323	65.78	9.72	1.02E-15	Bacteria	Firmicutes	Clostridia	Lachnospirales	Lachnospiraceae	Anaerosporobacter
ebc7527f35924613e64e071623af1abb	105.76	9.21	1.11E-10	Bacteria	Firmicutes	Clostridia	Lachnospirales	Lachnospiraceae	Anaerocolumna
148a7bc57792072043058510530ea32d	3855.11	5.58	9.73E-06	Bacteria	Firmicutes	Bacilli	Bacillales	Planococcaceae	Lysinibacillus
7406188bce39180c4aa194bfb387935e	37.30	6.73	1.75E-04	Bacteria	Firmicutes	Clostridia	Lachnospirales	Lachnospiraceae	Anaerostignum
d1e46966f512a55ca12a01802729f4ef	43.43	-6.04	7.07E-03	Bacteria	Proteobacteria	Gammaproteobacteria	Orbales	Orbaceae	Gilliamella
7d6f288bd50c0431d8fe542c02c1a0d2	14.62	6.40	1.93E-02	Bacteria	Firmicutes	Bacilli	Bacillales	Planococcaceae	Psychrobacillus
ffaedab25be5e2be043b2355be6644cf	17.78	7.61	2.53E-02	Bacteria	Firmicutes	Clostridia	Clostridiales	Clostridiaceae	Clostridium_sensu_stricto_1
05f5c262eb96bf78b6ab2750fdddc0c4	33.94	3.98	2.82E-02	Bacteria	Firmicutes	Clostridia	Lachnospirales	Lachnospiraceae	Lachnoclostridium
19019fa034a9856e8d6365d34574c8fd	544.72	-13.65	2.82E-02	Bacteria	Firmicutes	Bacilli	Lactobacillales	Leuconostocaceae	Weissella
c37413af5ea0a7254284c92b8bf54ae7	437.97	1.90	1.21E-01	Bacteria	Proteobacteria	Gammaproteobacteria	Enterobacterales	Morganellaceae	Providencia
9eadd1514e5fcfa10e723634e35f3485	377.67	2.43	1.45E-01	Bacteria	Actinobacteriota	Actinobacteria	Micrococcales	Microbacteriaceae	Microbacterium
0ff98df849b8fae49800a796dadc506c	93.90	1.33	3.02E-01	Bacteria	Firmicutes	Bacilli	Lactobacillales	Enterococcaceae	Enterococcus
4e817d860267844d0bb67bb3ed49522c	25.82	-2.10	3.12E-01	Bacteria	Firmicutes	Bacilli	Lactobacillales	Lactobacillaceae	Lactobacillus
b9a736421dc15f263c93ec9f509d5f6b	4.99	4.42	5.38E-01	Bacteria	Firmicutes	Clostridia	Peptostreptococcales-Tissierellales	Peptostreptococcaceae	Clostridioides
515ecf421a951bc274a7daaa791dc894	5.27	4.97	5.55E-01	Bacteria	Proteobacteria	Alphaproteobacteria	Rhodobacterales	Rhodobacteraceae	Paracoccus
f8d46ee1054ee9224a7e622fd2557cf4	7.49	2.51	6.58E-01	Bacteria	Firmicutes	Bacilli	Paenibacillales	Paenibacillaceae	Cohnella
7fda73c8ad009ec246fc6a5bf669203e	1638.56	-0.36	8.94E-01	Bacteria	Actinobacteriota	Actinobacteria	Corynebacteriales	Corynebacteriaceae	Corynebacterium
016b118f0377208f78e5694abe0d59b6	4.40	0.00	1.00E+00	Bacteria	Firmicutes	Clostridia	Oscillospirales	Ruminococcaceae	Incertae_Sedis
c2b73b7534124edd282fa67a545e048b	44.47	0.00	1.00E+00	Bacteria	Actinobacteriota	Actinobacteria	Corynebacteriales	Nocardiaceae	Rhodococcus
cd416ce5bf4afa614154fc0a45feb5a8	20.90	0.00	1.00E+00	Bacteria	Actinobacteriota	Actinobacteria	Micrococcales	Micrococcaceae	Glutamicibacter
c63d552898d6b61e6ef07797210d7aca	25.79	0.00	1.00E+00	Bacteria	Actinobacteriota	Actinobacteria	Micrococcales	Microbacteriaceae	Leucobacter
43f9d96deeccbd4b7c28e80612ec05ea	9.56	0.06	1.00E+00	Bacteria	Actinobacteriota	Actinobacteria	Micrococcales	Brevibacteriaceae	Brevibacterium
64bfc35722d08fe4ccf6b5665b9d1769	15.77	0.00	1.00E+00	Bacteria	Proteobacteria	Gammaproteobacteria	Enterobacterales	Morganellaceae	Morganella
5634c39a6b4a1475938b6ad7a4113445	23.37	0.00	1.00E+00	Bacteria	Firmicutes	Bacilli	Lactobacillales	Vagococcaceae	Vagococcus
2d5564533c7c3ef0e1f984107091c53c	1.05	0.00	1.00E+00	Bacteria	Firmicutes	Bacilli	Bacillales	Bacillaceae	Bacillus

baseMean: Mean abundance of the normalized count values over all samples included in the comparison; log2FC: log2-fold change represents the effect size estimate reported on a logarithmic scale to base 2, indicating how much the ASV abundance has changed due to selection for heat knockdown resistance in comparison to the control lines; *P* adj: adjusted P-values using the Benjamini-Hochberg procedure.

Table S8. Differential abundance analysis comparing lines selected for longevity (LS) with unselected control lines (UC) at genus level. Taxonomies were agglomerated to genus level prior to analysis. ASVs are ordered according to adjusted P-values (*P* adj) from lowest to highest. ASVs were considered differentially abundant for adjusted P-values ≤ 0.01 and an FDR cut-off of 5%. Significance threshold (*P* adj = 0.01) is marked by a dashed line.

ASV ID	baseMean	log2FC	*P* adj	Domain	Phylum	Class	Order	Family	Genus
810bf4f2547219682f5ab14a09ca3599	4949.58	-14.86	2.02E-21	Bacteria	Firmicutes	Bacilli	Lactobacillales	Leuconostocaceae	Leuconostoc
abc8f555ea0b005c9a4085a8343a3f99	326.35	9.53	3.80E-19	Bacteria	Firmicutes	Bacilli	Paenibacillales	Paenibacillaceae	Paenibacillus
19492e6d689a95489805fe62a81b5ecd	194.34	8.26	2.32E-18	Bacteria	Firmicutes	Bacilli	Brevibacillales	Brevibacillaceae	Brevibacillus
f8a687d17d5bcd547799c35ad359bf70	11655.70	6.59	5.78E-16	Bacteria	Proteobacteria	Alphaproteobacteria	Acetobacterales	Acetobacteraceae	Acetobacter
cd416ce5bf4afa614154fc0a45feb5a8	20.90	20.99	1.26E-14	Bacteria	Actinobacteriota	Actinobacteria	Micrococcales	Micrococcaceae	Glutamicibacter
33b3b41ddb67bb49f482abc6126da323	65.78	9.06	7.90E-14	Bacteria	Firmicutes	Clostridia	Lachnospirales	Lachnospiraceae	Anaerosporobacter
148a7bc577920720430585l0530ea32d	3855.11	8.27	1.06E-11	Bacteria	Firmicutes	Bacilli	Bacillales	Planococcaceae	Lysinibacillus
ebc7527f35924613e64e071623af1abb	105.76	7.63	1.13E-07	Bacteria	Firmicutes	Clostridia	Lachnospirales	Lachnospiraceae	Anaerocolumna
05f5c262eb96bf78b6ab2750fdddc0c4	33.94	7.84	8.66E-07	Bacteria	Firmicutes	Clostridia	Lachnospirales	Lachnospiraceae	Lachnoclostridium
9eadd1514e5fcfa10e723634e35f3485	377.67	6.01	2.93E-05	Bacteria	Actinobacteriota	Actinobacteria	Micrococcales	Microbacteriaceae	Microbacterium
7406188bce39180c4aa194bfb387935e	37.30	6.67	1.44E-04	Bacteria	Firmicutes	Clostridia	Lachnospirales	Lachnospiraceae	Anaerostignum
5634c39a6b4a1475938b6ad7a4113445	23.37	18.69	1.61E-03	Bacteria	Firmicutes	Bacilli	Lactobacillales	Vagococcaceae	Vagococcus
d1e46966f512a55ca12a01802729f4ef	43.43	-5.97	5.06E-03	Bacteria	Proteobacteria	Gammaproteobacteria	Orbales	Orbaceae	Gilliamella
19019fa034a9856e8d6365d34574c8fd	544.72	-14.26	1.91E-02	Bacteria	Firmicutes	Bacilli	Lactobacillales	Leuconostocaceae	Weissella
4e817d860267844d0bb67bb3ed49522c	25.82	-3.57	4.65E-02	Bacteria	Firmicutes	Bacilli	Lactobacillales	Lactobacillaceae	Lactobacillus
ffaedab25be5e2be043b2355be6644cf	17.78	6.43	5.18E-02	Bacteria	Firmicutes	Clostridia	Clostridiales	Clostridiaceae	Clostridium_sensu_stricto_1
c37413af5ea0a7254284c92b8bf54ae7	437.97	1.68	1.61E-01	Bacteria	Proteobacteria	Gammaproteobacteria	Enterobacterales	Morganellaceae	Providencia
f8d46ee1054ee9224a7e622fd2557cf4	7.49	5.11	1.93E-01	Bacteria	Firmicutes	Bacilli	Paenibacillales	Paenibacillaceae	Cohnella
7fda73c8ad009ec246fc6a5bf669203e	1638.56	-1.08	2.50E-01	Bacteria	Actinobacteriota	Actinobacteria	Corynebacteriales	Corynebacteriaceae	Corynebacterium
515ecf421a951bc274a7daaa791dc894	5.27	6.68	3.21E-01	Bacteria	Proteobacteria	Alphaproteobacteria	Rhodobacterales	Rhodobacteraceae	Paracoccus
0ff98df849b8fae49800a796dadc506c	93.90	0.86	5.15E-01	Bacteria	Firmicutes	Bacilli	Lactobacillales	Enterococcaceae	Enterococcus
7d6f288bd50c0431d8fe542c02c1a0d2	14.62	1.91	5.74E-01	Bacteria	Firmicutes	Bacilli	Bacillales	Planococcaceae	Psychrobacillus
016b118f0377208f78e5694abe0d59b6	4.40	3.52	6.56E-01	Bacteria	Firmicutes	Clostridia	Oscillospirales	Ruminococcaceae	Incertae_Sedis
b9a736421dc15f263c93ec9f509d5f6b	4.99	0.00	1.00E+00	Bacteria	Firmicutes	Clostridia	Peptostreptococcales-Tissierellales	Peptostreptococcaceae	Clostridioides
c2b73b7534124edd282fa67a545e048b	44.47	0.00	1.00E+00	Bacteria	Actinobacteriota	Actinobacteria	Corynebacteriales	Nocardiaceae	Rhodococcus
c63d552898d6b61e6ef07797210d7aca	25.79	0.00	1.00E+00	Bacteria	Actinobacteriota	Actinobacteria	Micrococcales	Microbacteriaceae	Leucobacter
43f9d96deeccbd4b7c28e80612ec05ea	9.56	0.34	1.00E+00	Bacteria	Actinobacteriota	Actinobacteria	Micrococcales	Brevibacteriaceae	Brevibacterium
64bfc35722d08fe4ccf6b5665b9d1769	15.77	0.00	1.00E+00	Bacteria	Proteobacteria	Gammaproteobacteria	Enterobacterales	Morganellaceae	Morganella
2d5564533c7c3ef0e1f984107091c53c	1.05	0.00	1.00E+00	Bacteria	Firmicutes	Bacilli	Bacillales	Bacillaceae	Bacillus

baseMean: Mean abundance of the normalized count values over all samples included in the comparison; log2FC: log2-fold change represents the effect size estimate reported on a logarithmic scale to base 2, indicating how much the ASV abundance has changed due to selection for longevity in comparison to the control lines; P adj: adjusted P-values using the Benjamini-Hochberg procedure.

Table S9. Differential abundance analysis comparing lines selected for starvation resistance (SS) to the unselected control lines (UC) at the genus level. Taxonomies were agglomerated to genus level prior to analysis. ASVs are ordered according to adjusted P-values (*P* adj) from lowest to highest. ASVs were considered differentially abundant for adjusted P-values ≤ 0.01 and an FDR cut-off of 5%. Significance threshold (*P* adj = 0.01) is marked by a dashed line.

ASV ID	baseMean	log2FC	*P* adj	Domain	Phylum	Class	Order	Family	Genus
abc8f555ea0b005c9a4085a8343a3f99	326.35	10.15	2.16E-21	Bacteria	Firmicutes	Bacilli	Paenibacillales	Paenibacillaceae	Paenibacillus
cd416ce5bf4afa614154fc0a45feb5a8	20.90	22.85	6.69E-17	Bacteria	Actinobacteriota	Actinobacteria	Micrococcales	Micrococcaceae	Glutamicibacter
810bf4f2547219682f5ab14a09ca3599	4949.58	-8.84	1.10E-09	Bacteria	Firmicutes	Bacilli	Lactobacillales	Leuconostocaceae	Leuconostoc
ebc7527f35924613e64e071623af1abb	105.76	8.35	9.52E-09	Bacteria	Firmicutes	Clostridia	Lachnospirales	Lachnospiraceae	Anaerocolumna
33b3b41ddb67bb49f482abc6126da323	65.78	7.09	1.15E-08	Bacteria	Firmicutes	Clostridia	Lachnospirales	Lachnospiraceae	Anaerosporobacter
19492e6d689a95489805fe62a81b5ecd	194.34	4.84	7.36E-07	Bacteria	Firmicutes	Bacilli	Brevibacillales	Brevibacillaceae	Brevibacillus
05f5c262eb96bf78b6ab2750fdddc0c4	33.94	7.76	1.44E-06	Bacteria	Firmicutes	Clostridia	Lachnospirales	Lachnospiraceae	Lachnoclostridium
c37413af5ea0a7254284c92b8bf54ae7	437.97	4.91	3.23E-06	Bacteria	Proteobacteria	Gammaproteobacteria	Enterobacterales	Morganellaceae	Providencia
7406188bce39180c4aa194bfb387935e	37.30	7.31	3.00E-05	Bacteria	Firmicutes	Clostridia	Lachnospirales	Lachnospiraceae	Anaerostignum
5634c39a6b4a1475938b6ad7a4113445	23.37	24.09	3.14E-05	Bacteria	Firmicutes	Bacilli	Lactobacillales	Vagococcaceae	Vagococcus
c2b73b7534124edd282fa67a545e048b	44.47	9.02	2.06E-04	Bacteria	Actinobacteriota	Actinobacteria	Corynebacteriales	Nocardiaceae	Rhodococcus
9eadd1514e5fcfa10e723634e35f3485	377.67	4.95	6.65E-04	Bacteria	Actinobacteriota	Actinobacteria	Micrococcales	Microbacteriaceae	Microbacterium
c63d552898d6b61e6ef07797210d7aca	25.79	8.31	7.92E-04	Bacteria	Actinobacteriota	Actinobacteria	Micrococcales	Microbacteriaceae	Leucobacter
148a7bc57792072043058510530ea32d	3855.11	3.55	5.57E-03	Bacteria	Firmicutes	Bacilli	Bacillales	Planococcaceae	Lysinibacillus
19019fa034a9856e8d6365d34574c8fd	544.72	-15.51	8.94E-03	Bacteria	Firmicutes	Bacilli	Lactobacillales	Leuconostocaceae	Weissella
4e817d860267844d0bb67bb3ed49522c	25.82	-4.14	1.55E-02	Bacteria	Firmicutes	Bacilli	Lactobacillales	Lactobacillaceae	Lactobacillus
7d6f288bd50c0431d8fe542c02c1a0d2	14.62	5.98	1.89E-02	Bacteria	Firmicutes	Bacilli	Bacillales	Planococcaceae	Psychrobacillus
f8a687d17d5bcd547799c35ad359bf70	11655.70	-1.76	4.29E-02	Bacteria	Proteobacteria	Alphaproteobacteria	Acetobacterales	Acetobacteraceae	Acetobacter
d1e46966f512a55ca12a01802729f4ef	43.43	-4.18	4.62E-02	Bacteria	Proteobacteria	Gammaproteobacteria	Orbales	Orbaceae	Gilliamella
0ff98df849b8fae49800a796dadc506c	93.90	1.40	2.09E-01	Bacteria	Firmicutes	Bacilli	Lactobacillales	Enterococcaceae	Enterococcus
ffaedab25be5e2be043b2355be6644cf	17.78	4.03	2.35E-01	Bacteria	Firmicutes	Clostridia	Clostridiales	Clostridiaceae	Clostridium_sensu_stricto_1
43f9d96deeccbd4b7c28e80612ec05ea	9.56	-3.50	2.97E-01	Bacteria	Actinobacteriota	Actinobacteria	Micrococcales	Brevibacteriaceae	Brevibacterium
f8d46ee1054ee9224a7e622fd2557cf4	7.49	3.02	4.53E-01	Bacteria	Firmicutes	Bacilli	Paenibacillales	Paenibacillaceae	Cohnella
2d5564533c7c3ef0e1f984107091c53c	1.05	4.60	4.84E-01	Bacteria	Firmicutes	Bacilli	Bacillales	Bacillaceae	Bacillus
64bfc35722d08fe4ccf6b5665b9d1769	15.77	1.51	6.51E-01	Bacteria	Proteobacteria	Gammaproteobacteria	Enterobacterales	Morganellaceae	Morganella
b9a736421dc15f263c93ec9f509d5f6b	4.99	0.00	1.00E+00	Bacteria	Firmicutes	Clostridia	Peptostreptococcales-Tissierellales	Peptostreptococcaceae	Clostridioides
016b118f0377208f78e5694abe0d59b6	4.40	0.00	1.00E+00	Bacteria	Firmicutes	Clostridia	Oscillospirales	Ruminococcaceae	Incertae_Sedis
7fda73c8ad009ec246fc6a5bf669203e	1638.56	0.09	1.00E+00	Bacteria	Actinobacteriota	Actinobacteria	Corynebacteriales	Corynebacteriaceae	Corynebacterium
515ecf421a951bc274a7daaa791dc894	5.27	0.00	1.00E+00	Bacteria	Proteobacteria	Alphaproteobacteria	Rhodobacterales	Rhodobacteraceae	Paracoccus

baseMean: Mean abundance of the normalized count values over all samples included in the comparison; log2FC: log2-fold change represents the effect size estimate reported on a logarithmic scale to base 2, indicating how much the ASV abundance has changed due to selection for starvation resistance in comparison to the control lines; *P* adj: adjusted P-values using the Benjamini-Hochberg procedure.
